# Structural Diversity and Dynamics of Human Three-Finger Proteins Acting on Nicotinic Acetylcholine Receptors

**DOI:** 10.3390/ijms21197280

**Published:** 2020-10-01

**Authors:** Alexander S. Paramonov, Milita V. Kocharovskaya, Andrey V. Tsarev, Dmitrii S. Kulbatskii, Eugene V. Loktyushov, Mikhail A. Shulepko, Mikhail P. Kirpichnikov, Ekaterina N. Lyukmanova, Zakhar O. Shenkarev

**Affiliations:** 1 Shemyakin-Ovchinnikov Institute of Bioorganic Chemistry, Russian Academy of Sciences, 119997 Moscow, Russia; a.s.paramonov@gmail.com (A.S.P.); kocharovskaya.mv@phystech.edu (M.V.K.); tsarev2709@yandex.ru (A.V.T.); d.kulbatskiy@gmail.com (D.S.K.); zhenyaloktushov@mail.ru (E.V.L.); mikhailshulepko@gmail.com (M.A.S.); kirpichnikov@inbox.ru (M.P.K.); 2 Phystech School of Biological and Medical Physics, Moscow Institute of Physics and Technology (National Research University), 141701 Dolgoprudny, Moscow Region, Russia; 3 Faculty of Biology, Lomonosov Moscow State University, 119234 Moscow, Russia

**Keywords:** Ly-6/uPAR, three-finger proteins, nicotinic acetylcholine receptors, NMR spectroscopy, ^15^N-relaxation, backbone dynamics

## Abstract

Ly-6/uPAR or three-finger proteins (TFPs) contain a disulfide-stabilized β-structural core and three protruding loops (fingers). In mammals, TFPs have been found in epithelium and the nervous, endocrine, reproductive, and immune systems. Here, using heteronuclear NMR, we determined the three-dimensional (3D) structure and backbone dynamics of the epithelial secreted protein SLURP-1 and soluble domains of GPI-anchored TFPs from the brain (Lynx2, Lypd6, Lypd6b) acting on nicotinic acetylcholine receptors (nAChRs). Results were compared with the data about human TFPs Lynx1 and SLURP-2 and snake α-neurotoxins WTX and NTII. Two different topologies of the β-structure were revealed: one large antiparallel β-sheet in Lypd6 and Lypd6b, and two β-sheets in other proteins. α-Helical segments were found in the loops I/III of Lynx2, Lypd6, and Lypd6b. Differences in the surface distribution of charged and hydrophobic groups indicated significant differences in a mode of TFPs/nAChR interactions. TFPs showed significant conformational plasticity: the loops were highly mobile at picosecond-nanosecond timescale, while the β-structural regions demonstrated microsecond-millisecond motions. SLURP-1 had the largest plasticity and characterized by the unordered loops II/III and *cis-trans* isomerization of the Tyr39-Pro40 bond. In conclusion, plasticity could be an important feature of TFPs adapting their structures for optimal interaction with the different conformational states of nAChRs.

## 1. Introduction

Ly-6/uPAR proteins or ‘three-finger’ proteins (TFPs) contain characteristic ‘three-finger’ domain(s) or LU-domain(s) [1]. Each LU-domain is composed of a compact disulfide-stabilized β-structural core (‘head’) and three protruding loops (‘fingers’). To date, TFPs have been found in *Asterozoa* [2], *Arthropoda* [3], and *Vertebrata* including fishes, amphibians, reptiles, birds, and mammals [1]. The best-studied TFPs are snake neurotoxins and the receptor of urokinase-type plasminogen activator (uPAR), containing one and three LU-domains, respectively. There are 48 TFPs genes in the human genome [4]. These proteins were found in the nervous, endocrine, reproductive, and immune systems and in the epithelium.

There are many regulatory TFPs containing the single LU-domain. These proteins may be classified into secreted (e.g., SLURP-1 and SLURP-2) and GPI-anchored (e.g., Lynx1, Lynx2, Lypd6, and Lypd6b) proteins. For some TFPs (e.g., Lynx1, PCSA, CD59, uPAR), both soluble and membrane-tethered forms were reported [5,6,7,8]. The secreted Lynx2 isoform was also predicted due to presence of a potential proteolytic cleavage site between the LU-domain and GPI anchor [9].

TFPs target various types of membrane receptors [1], but many of them are able to interact with nicotinic acetylcholine receptors (nAChRs). For example, Lynx1 is colocalized with α7- and α4β2-nAChRs in the brain [10] and modulates their function [11], in particular controlling an assembly of α4β2-nAChRs in the endoplasmic reticulum [12]. Lynx1 regulates neuronal plasticity during postnatal development [13], motor learning [14], and other cognitive functions by interaction with α7-nAChRs [15]. Similarly, Lynx2 (also known as LYPD1) is expressed in the peripheral and central nervous systems [16] and modulates α4β2-nAChRs [17], probably controlling their assembly [18]. Lynx2-KO mice demonstrated reduced anxiety level [17]. Despite having similar pharmacological properties, expression profiles of the *Lynx1* and *Lynx2* genes in the rodent brain and during the organism development in the postnatal period are significantly different [6,17]. Besides the brain, *Lynx1* and *Lynx2* expression was also detected in a lung tissue [19]. The Lynx1 protein was also found in different epithelial cells [20], where it modulates a non-neuronal cholinergic signaling and controls cell proliferation and apoptosis [20,21].

Lypd6 and Lypd6b were found in the cerebral cortex, spinal cord, and other tissues (lung, kidneys, heart, liver, testes, stomach, prostate) [22,23,24,25]. Lypd6 participates in a formation of the Wnt/β-catenin receptor complex [26] and interacts with the Wnt coreceptor LRP6 [27], while Lypd6b probably participates in the protein kinase C (PKC) signal transduction pathway increasing the pro-transcriptional activity of phorbol myristate acetate (PMA) [25]. Both proteins also target nAChRs. Lypd6 overexpression leads to an increased locomotor activity and visceral hyperalgesia, typical for increased cholinergic tone, and to enhanced nicotine-induced calcium currents amplitude in trigeminal ganglion neurons of transgenic mice [22]. Lypd6-KO mice demonstrated significantly increased amplitude of nicotine-induced currents in dorsal raphe nuclei and reduced anxiety-like behavior [28]. Recombinant Lypd6 fused with glutathione-S-transferase inhibits a nicotine-induced current in the CA1 region of hippocampus and is able to extract the α3, α4, α5, α6, α7, β2, and β4 nAChR subunits from the human brain homogenate [23]. Lypd6b interaction with α3β4-, but not with α7-nAChR, was described [29].

In contrast to GPI-anchored proteins from the nervous system, SLURP-1 and SLURP-2 are secreted auto/paracrine regulators of epithelial cells homeostasis [30]. The main target of SLURP-1 in epithelial cells is α7-nAChR [31], while SLURP-2 can act on different nAChR subtypes and on muscarinic acetylcholine receptors (mAChRs) [32]. These TFPs control growth, differentiation, migration, and apoptosis of epithelial cells, as well as skin and mucous wound healing [33,34] and development of inflammation and tumors [35,36,37]. SLURP-1 expression is down-regulated in primary melanomas and to more extent in metastatic melanomas compared to normal cells [38]. Mutations in the *SLURP1* gene cause a recessively inherited skin disease palmoplantar keratoderma known as Mal de Meleda (OMIM 248300) [39]. The similar disease phenotype is developed in the SLURP-2-KO mice [40].

Data about the structure of human TFPs and mode of their interaction with the target receptors are needed for *in silico* drug design. Currently, most human TFPs acting on nAChRs remain poorly studied. Only one crystal structure of the Lypd6 LU-domain [27] and two NMR structures of SLURP-2 and the Lynx1 LU-domain were published [11,32]. Here, we have tried to fill this gap and describe the NMR structures of four human TFPs: Lynx2, Lypd6, Lypd6b, and SLURP-1. We focused not only on the structural properties, but also on the dynamics and conformational plasticity of the proteins. The data about motions at various time scales obtained from ^15^N-relaxation were compared with the results of NMR structure calculations and, in particular, with the precision of the NMR structural ensembles in different regions of the three-finger structure. Obtained data, in comparison with the results of previous NMR studies of Lynx1, SLURP-2, and three-finger snake toxins, revealed significant differences in the structural and dynamic properties of the three-finger proteins. Relationships between the structure, dynamics, and function of the Ly6/uPAR proteins are discussed.

## 2. Results

### 2.1. Design, Production, and Optimization of Conditions for NMR Study of Isolated LU-Domains

**Figure 1:**
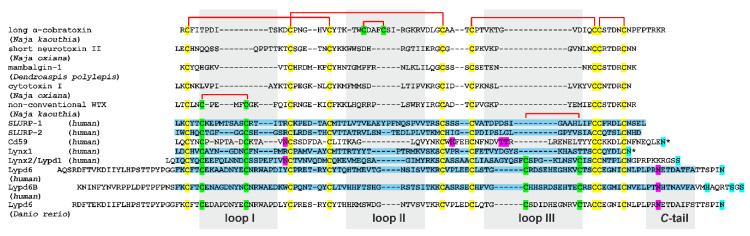
Amino acid sequence alignment of three-finger proteins (TFPs). Representative snake toxins from various families, several human TFPs, and fish Lypd6 are shown. The signal peptides are removed. Invariant Cys residues are shown in yellow. Cys residues forming additional disulfide bonds in the loops I, II, and III are shown in green. Disulfide bonds are shown by red brackets. Predicted sites for attachment of the GPI-anchor and glycosylation are shown by cyan and magenta, respectively. Several possible GPI-anchor sites were predicted for human Lypd6b. Fragments of the proteins used for NMR studies in this and previous works [11] are highlighted in blue. The proteins, for which the presence of both the GPI-anchored and soluble form were reported, are marked by an asterisk. The loop regions and *C*-terminal regions used for calculation of mean RMSD and S^2^ values are highlighted by gray background. PDB codes: α-cobratoxin—2CTX, neurotoxin II—2MJ4, mambalgin-1—5DU1, cytotoxin I—5NPN, WTX—2MJ0, SLURP-1—6ZZE/6ZZF, SLURP-2—2N99, CD59—2J8B, Lynx1—2L03, Lynx2—6ZSS, Lypd6—6IB6, Lypd6B—6ZSO. The structure of *Danio rerio* Lypd6 is not available.

Isotopically (^13^C, ^15^N) labeled variants of the human SLURP-1, Lypd6, Lypd6b, and Lynx2 proteins were produced using a previously developed approach [41]. The *SLURP-1* expression construct encoded the full-length protein without signal peptide (Leu1(23)-Asn81(103), where the numbers in brackets correspond to residue numbers in the protein starting from the signal peptide. Unlike the other TFPs containing single LU-domain, Lypd6 and Lypd6b have prolonged *N*- and *C*-terminal sequences enclosing the three-finger domain (Figure 1). These fragments were excluded from the expression constructs because they prevented correct folding of the recombinant proteins [42]. Therefore, the *Lypd6* and *Lypd6b* constructs encoded Phe1(47)-Ala95(141) and Phe1(60)-Ala95(154) fragments, respectively, and did not include the *C*-terminal sites for attachment of a GPI-anchor (Figure 1). *Lynx2* expression construct encoded the LU-domain (Ile1(23)-Asn85(107)) without the signal peptide, two *N*-terminal residues (Leu21-Gln22), and *C*-terminal site for attachment of a GPI-anchor (Ser95(117)) (Figure 1). Each recombinant TFP contained an additional *N*-terminal Met residue, which appeared due to translation of the starting *atg* codon and numbered as Met0.

Pairwise alignment of the TFPs sequences using CLUSTAL W2 software (https://www.ebi.ac.uk/Tools/msa/clustalw2/) revealed a relatively high similarity among the proteins of various origins. The least similarity (20%) was observed between Lynx1 and cobra non-conventional toxin WTX. The greatest similarity among the proteins studied in this work was found between Lypd6 and Lypd6b (60%) and SLURP-1 and SLURP-2 (40%). The similarities between Lynx2 and WTX, SLURP-1 and short toxin NTII, Lypd6b and Lynx2, and SLURP-2 and Lynx2 were found to be 37, 36, 36, and 31%, respectively.

The proteins studied here have different physicochemical properties: overall charge, hydrophobicity, and pI value (Table 1). Therefore, to optimize the sample stability and quality of NMR spectra, we varied sample pH and temperature of NMR measurements. The optimization of pH is critical for NMR structure determination. On the one hand, the use of pH values close to the pI value of the protein generally should be avoided, because this can lead to protein aggregation, especially at high concentrations required for NMR. On the other hand, a weakly acidic pH (4.5–5.5) is beneficial for observation of signals from flexible and disordered regions of the proteins due to minimization of the exchange of HN protons with water and increased sensitivity of NMR experiments. The Asp and Glu sidechains and *C*-terminal carboxyl group of the proteins usually have pKa values less than 4.5 and, therefore, their ionization state does not change when the pH changes from 7.0 to 5.0, and they remain charged in weakly acidic conditions. At the same time, the His sidechains become protonated and acquire a positive charge. The change in the ionization state of some structurally important residues can significantly influence the protein conformation. For example, we observed previously the reversible unfolding of Lynx1 and SLURP-2 at pH below 4.5 [11,32]. It should be noted that, to a first approximation, a variation of the solvent pH in the range 3–8 does not affect the backbone-backbone hydrogen bonds and bonds with non-ionizable sidechains (Asn, Gln, Ser, Thr, Tyr, Trp) in proteins.

We qualitatively monitored the preservation of the protein fold and absence of aggregation by two-dimensional (2D) ^15^N-HSQC spectra. Here and in the previous study [42], we have observed that the Lypd6b protein does not lose its structure and has sufficient stability at pH 5.5, lower than its pI value (6.7). At the same time, the Lypd6 and Lynx2 proteins, which both have a pI of about 5, could not withstand a decrease in pH, which led to a very poor quality of the HSQC spectra. Therefore, the optimal pH for the study of these proteins was about 7 (Table 1). Surprisingly, the SLURP-1 protein demonstrated the relatively good stability and preserved the three-finger fold at pH 4.7, only slightly lower than its pI value (5.2) [46]. It should be noted that SLURP-1 is the only truly soluble protein in our study; other proteins are soluble domains of the membrane bound molecules. Most likely, SLURP-1 is well suited to maintain solubility at high concentrations in epithelial tissues, where pH varies over a wide range from 4.5 to 7.0 [47].

The temperature choice for the NMR measurements was also made by optimizing the quality of the 2D ^15^N-HSQC spectra. The linear temperature dependencies of ^1^H^N^ chemical shifts confirmed the absence of significant structural changes in the studied proteins in the temperature range 20–45 °C (Figure S1A–D).

### 2.2. 3D Structures of Human TFPs

**Figure 2:**
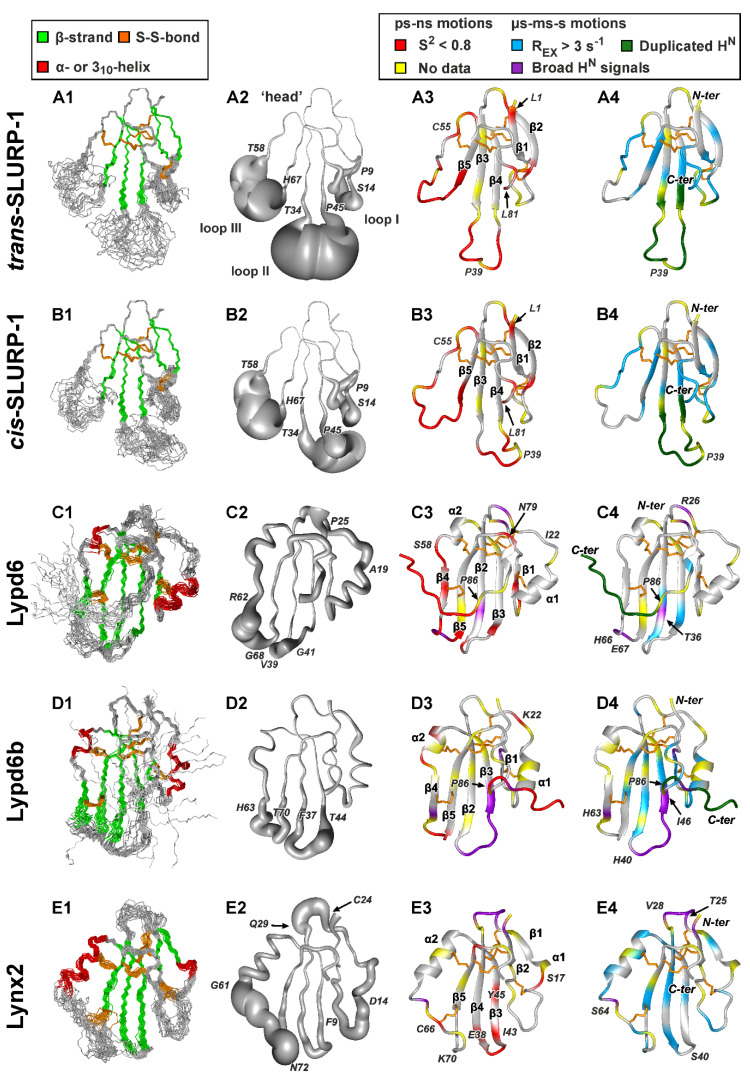

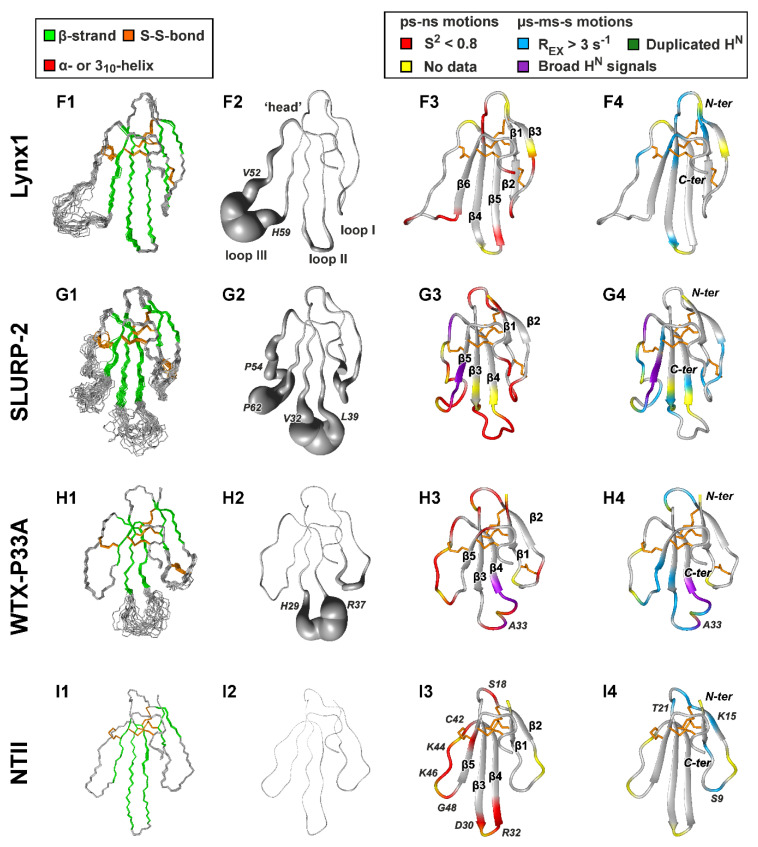
NMR structures and dynamics of TFPs: (**A**) *trans*-Tyr39-Pro40 SLURP-1, (**B**) *cis*-Tyr39-Pro40 SLURP-1, (**C**) Lypd6, (**D**) Lypd6b, (**E**) Lynx2, (**F**) Lynx1, (**G**) SLURP-2, (**H**) WTX-P33A, (**I**) NTII. For each protein/isomer, four panels are shown. (1) Set of the 20 best CYANA structures. Protein backbone and disulfide bonds (gold) are shown. Secondary structure elements are color-coded: red—α- or 3_10_-helix, green—β-sheet. (2) ‘Sausage’ representation of the mean structure with variable radius equal to the average displacement in the set of structures. In the Lypd6 and Lypd6b structures, the completely disordered *C*-terminal fragments (Leu85-Ala95) are omitted. (3–4). Ribbon representation of NMR structures with mapped data about ‘fast’ (ps–ns, 3) and ‘slow’ (µs-ms-s, 4) backbone dynamics. Regions with the high-amplitude ps–ns mobility (generalized order parameter S^2^ < 0.8) are in red. Sites of µs-ms-s conformational fluctuations are in blue (exchange contributions to R_2_ relaxation rates R_ex_ > 3 s^−1^ at 800 MHz), violet (significant broadening of ^1^H^N^ signals), and dark green (two protein conformers were observed). *N*-terminal Met0 residue, Pro residues, and the residues for which dynamics data are unavailable due to spectral overlap are highlighted by yellow. The residues from the disordered *C*-terminal fragments of Lypd6 and Lypd6b, which did not satisfactorily fit into any model of intramolecular mobility, are shown in red. Structural data for Lynx1, SLURP-2, WTX-P33A, and NTII were taken from previous publications [11,32,43,44]; other data are from the present work. PDB codes: WTX-P33A—2MJ0, SLURP-1—6ZZE/6ZZF, SLURP-2—2N99, Lynx1—2L03, Lynx2—6ZSS, Lypd6—6IB6, Lypd6B—6ZSO.

Almost complete backbone and side chain ^1^H, ^13^C, ^15^N resonance assignment was obtained for SLURP-1, Lypd6, Lypd6b, and Lynx2 (Table S1). Three-dimensional (3D) structures of the proteins were calculated using NMR data (see Materials and Methods), summarized in Figure S2A–D. Statistics of the calculated sets of structures is collected in Table S4. In all cases, the three-finger fold with the compact disulfide-stabilized ‘head’ and three less ordered loops was observed (Figure 2).

#### 2.2.1. SLURP-1

In addition to four invariant disulfide bonds stabilizing the ‘head’ (Cys3-Cys28, Cys21-Cys51, Cys55-Cys71, and Cys72-Cys77), SLURP-1 contains fifth disulfide in the loop I (Cys6-Cys15). Two sets of signals corresponding to the two equally populated structural states have been previously observed in the NMR spectra of SLURP-1 [46]. This conformation heterogeneity is caused by *cis-trans* isomerization of the Tyr39-Pro40 peptide bond located in the tip of the loop II. The isomerization process goes with characteristic time~4 ms [46]. Here, using a double labeled (^13^C, ^15^N) sample, we obtained complete resonance assignment (Figure S3) and determined 3D structures of both (*trans-* and *cis-*) SLURP-1 isomers (Figure 2A,B). Secondary structure of the both isomers is formed by two antiparallel β-sheets. The first β-sheet has two strands and involves residues from the loop I (Leu1-Thr5 and Thr17-Cys21). The second β-sheet consists of three strands and includes fragments of the loop II (Ala27-Val33, Val46-Ser52) and loop III (Leu68-Cys72). Like other TFPs, the SLURP-1 molecule contains conserved β-turns in the ‘head’ (Lys22-Asp25) and *C*-terminus (Arg74-Cys77, Figure S2A). The spatial arrangement of *N*- and *C*-termini is controlled by H^N^ Lys2–O^δ1^ Asp75 and H^N^ Leu76–CO Lys2 hydrogen bonds. The first of these bonds involve ionizable sidechain of Asp75, so we can expect that a decrease in pH well below 4.5 could lead to disruption of the interaction between *N*- and *C*-termini and could potentially result in the protein unfolding, as was previously observed for Lynx1 and SLURP-2 [11,32].

**Figure 3:**
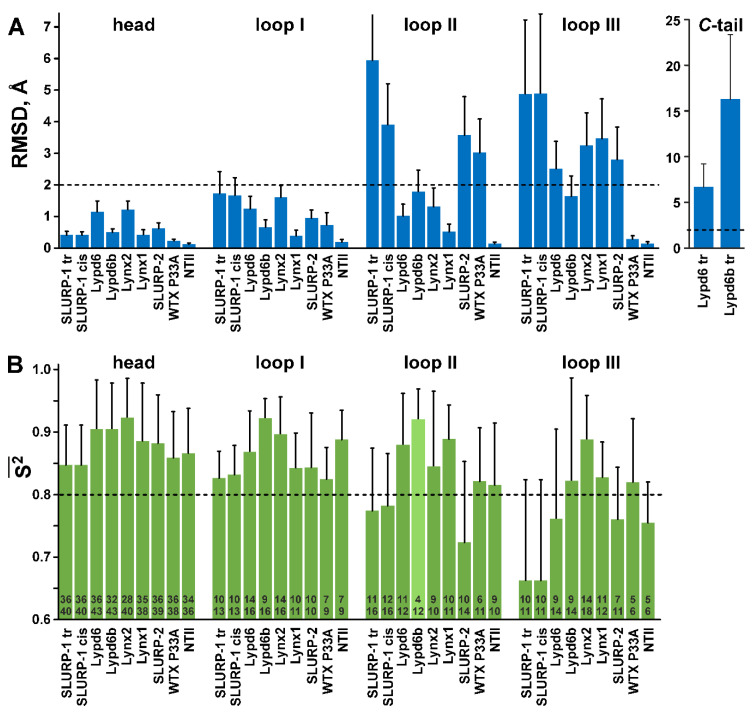
Qualitative comparison of NMR structure precision (**A**) with amplitude of ps–ns dynamics (**B**). (**A**). Mean backbone RMSD values (Å) in the NMR sets of the TFPs structures calculated over the loop regions, *C*-terminal ‘tail,’ and ‘head’ (other residues). The boundaries of the loop and *C*-terminal regions in the protein sequences are shown in Figure 1 with a gray background. Threshold value (RMSD > 2.0 Å, dashed line) shows the protein fragments with significant disorder. (**B**). Mean generalized order parameters (S^2^) calculated over the loop regions, and ‘head’ of TFPs. The calculation of S^2^ values in the *C*-terminal ‘tails’ was impossible, probably due to a very complex mobility model of the corresponding residues, which cannot be adequately described by models ##1–5. The threshold value (S^2^ < 0.8, dashed line) shows the protein fragments with high-amplitude ps–ns dynamics. The numbers within the bars denote the length of the corresponding region (lower number) and number of residues with ^15^N relaxation data available (upper number). The mean S^2^ value in the Lypd6b loop II (light green) has the large degree of uncertainty due to lack of experimental data.

The tip regions of the loops II (Thr34-Pro45) and III (Thr58-His67) were not converged to any defined conformation (Figure 2(A2,B2)). Lack of the long-range NOE-contacts, values of ^3^J_H_^N^_H_^α^ couplings, and chemical shift data confirmed the absence of specific conformation in these regions (Figure S2A). A lesser degree of disorder was observed at the tip of the loop I (Pro9-Ser14) and *C*-terminus (Ser79-Leu81), while other regions of the protein were well defined. The degree of disorder in the SLURP-1 loops is illustrated by RMSD calculations (Figure 3A). Loop I in both isomers was characterized by mean RMSD ~1.7 Å, while RMSD of the loops II and III exceeded 3.0 Å. At the same time, RMSD values in the ‘head’ region were ~0.4 Å. We should mention here that the plasticity of different TFPs on the per residue basis was additionally analyzed using ^15^N relaxation data (see Section 2.3 of the manuscript and Figure 3B). The relaxation analysis does not depend on the quality of the 3D structure determination and gives more reliable estimates of the structure plasticity (see below).

Analysis of the 3D structures of *trans-* and *cis-*SLURP-1 isomers revealed no significant differences in the ordered regions. Contrary, the chemical shifts, ^3^J_H_^N^_H_^α^, and Δδ^1^H^N^/ΔT values pointed to the different conformation of disordered regions in the loops I and II (Figure S2A). Moreover, the *trans-* and *cis-*isomers had different propensity of the β-structure formation (P_β_) in the loop II. Thus, the *cis-trans* isomerization of the Tyr39-Pro40 peptide bond influenced the conformation of β3- and β4-strands in the loop II and induced the conformational changes in the loop I.

#### 2.2.2. Lypd6 and Lypd6b

The Lypd6 and Lypd6b proteins have six disulfide bonds: four invariant bonds in the ‘head’ (Cys3-Cys31, Cys24-Cys50, Cys56-Cys75, and Cys76-Cys81) and two additional disulfides in the loops I and III (Cys6-Cys15 and Cys61-Cys72). The preliminary NMR study of the Lypd6 and Lypd6b LU-domains [42] revealed conformation heterogeneity in the *C*-terminal regions (Pro86-Ala95) probably associated with *cis-trans* isomerization of the Leu85-Pro86 peptide bond. Two isomers of Lypd6 were equally populated, and exchange between them was relatively slow (characteristic time > 0.2 s), while some resonances of the Lypd6b *cis-*form were significantly broadened indicating faster exchange rate. NMR data analysis (Figure S2B,C) revealed that the *C*-termini in the both Lypd6 isomers and in the Lypd6b *trans-*isomer are disordered.

The 3D structures of the *trans*- Lypd6 and Lypd6b isomers were calculated (Figure 2C,D). In contrast to previously studied TFPs, the Lypd6 and Lupd6b molecules contain only one wide antiparallel β-sheet formed by five strands. The first short β1 strand involves three or two residues of the loop I (Cys6-Lys8/Cys6-Glu7, for Lypd6/Lypd6b, respectively). Three central β2, β3, and β5 strands are significantly longer and include fragments of the loops II and III (Tyr30-Glu38/Tyr30-Thr38, Ser43-Val51/Ser45-Ala51, and His69-Cys76/His69-Cys76, for Lypd6/Lypd6b, respectively). Another short β4 strand involves three or five residues of the loop III (Gly60-Arg62/Gly60-Ser64, for Lypd6/Lypd6b, respectively). The α-helical element α1 was observed in the loop I (Asn12-Trp18/Asn12-Ala19), and short helical turn α2 (Leu53-Cys56/Arg53-His57) was found in the ‘head’ of Lypd6/Lypd6b (Figure 2C,D).

Like other TFPs, Lypd6 and Lypd6b contain β- and γ-turns (Ile80-Leu83/Gly78-Val83, respectively) at the *C*-termini of the LU-domains (Figure S2B,C). Spatial arrangement of the *N*- and *C*-termini of the LU-domains is controlled by two hydrogen bonds (H^N^ Ile80–CO Lys2 and H^Nδ^ Asn82–CO Phe4). At the same time, the conserved β-turn in the ‘head’ region was observed only in the Lypd6b (Pro25-Thr28). The Lypd6 structure has the additional tight γ-turn (Pro20-Ile22) in the loop I after helix α1. Interestingly, the tip of the Lypd6 loop II (Glu38-Gly41) adopts β-turn conformation, which together with the β2 and β3 strands forms prolonged β-hairpin (Figure 2C). In contrast, the Lypd6b loop II is less ordered due to significant resonance broadening (Figure 2D).

In the calculated set of structures (Figure 2(C1,D1)), significant disorder was observed in the *C*-terminal ‘tail’ (Leu85-Ala95) and in the tips of the loop II (Val39-Gly41/Phe37-Thr44) and loop III (Arg62-Gly68/His63-Thr70) for Lypd6/Lypd6b, respectively. In addition, the three *N*-terminal Lypd6 residues (Met0-Lys2) and the neighboring fragment of the loop I (Ala19-Pro25) were weakly structured (Figure 2(C2)). The large RMSD values exceeding 2.0 Å were observed in the Lypd6 loop III and in the *C*-terminal ‘tails’ of both proteins (Figure 3A).

Recently, the crystal structure of the Lypd6 LU-domain was published [27]. The crystallized protein contained, besides the fragment (Phe1(47)-Ala95(141)), additional *N*- and *C*-terminal sequences with purification tags and protease cleavage site. Comparison of the crystal and solution structures of the Lypd6 LU-domain revealed quite good correspondence (Figure S4), but the *C*-terminus conformations were different. Only *trans*-isomer of Lypd6 was observed in the crystal, and the *C*-terminal ‘tail’ was well ordered, bent towards the protein β-structural core, and formed additional β6 strand. Analysis of the crystal packing revealed that, in neighboring asymmetric units, the *C*-terminal ‘tails’ of two Lypd6 molecules are packed against each other with interatomic distances in the range 2–3 Å (Figure S5 and Table S3). Thus, crystallization overstabilized the protein by formation of the artificial intramolecular and intermolecular contacts.

#### 2.2.3. Lynx2

Preliminary NMR characterization of the Lynx2 LU-domain [48] revealed significant broadening of the backbone ^1^H^N^ resonances at 30 °C (Figure S6A). The degree of broadening was diminished with the temperature increase, and the spectra quality became acceptable at 45 °C. Nevertheless, even at elevated temperatures, the Thr25-Val28 (‘head’) and Ser64 (loop III) residues remain unobservable (Figure 2(E4), violet, and Figure S2D, underlined). Comparison of 2D ^15^N-HSQC Lynx2 spectra measured at 30 °C and 45 °C did not reveal significant changes in the protein structure (Figure S6A,B). Thus, the observed broadening of NMR signals was due to the exchange process at the µs-ms timescale. This process was in fast-to-intermediate mode at 30 °C and switched to the fast mode at 45 °C. ^15^N-relaxation data measured at several Lynx2 concentrations (Figure S3D) revealed that this process originated by the protein oligomerization in solution.

The 3D structure of Lynx2 was calculated using data measured at 45 °C for the sample with 0.25 mM concentration (Figure 2E). The samples with lower concentrations did not provide sufficient quality of the structural data. Lynx2 molecule is stabilized by six disulfide bonds: four invariant bonds in the ‘head’ (Cys3-Cys32, Cys24-Cys49, Cys55-Cys78, and Cys79-Cys84) and two additional disulfides in the loops I and III (Cys6-Cys15 and Cys66-Cys75). The Lynx2 β-structure involves two antiparallel β-sheets with arrangement similar to SLURP-1 (Figure 2E). The first β-sheet in the loop I is formed by two strands (Gln2-Tyr4 and Ile21-Asn23). The second β-sheet consists of three strands and includes fragments of the loop II (Met31-Gln39, Gly42-Ala50) and loop III (Cys75-Cys79). Similar to Lypd6/Lypd6b, Lynx2 contains two helical elements in the loop I and III, but the length of the helices was different. One turn of 3_10_-helix (α1) was observed in the loop I (Pro18-Phe20), and long α2-helix (Ser51-Gly61) extends from the loop III to the ‘head’ of the protein (Figure 2E). Due to exchange-induced broadening of NMR resonances, the conformation of the Cys24-Gln29 loop in the Lynx2 ‘head’ remains undefined. This made the detection of β- or γ-turns in the corresponding region impossible. Nevertheless, the tight β-turn was observed in the tip of the loop II (Gln39-Gly42), which together with the β3 and β4 strands forms β-hairpin (Figure 2E). Conserved β-turn (Thr81-Cys84) and hydrogen bonds (H^N^ Leu83–CO Lys2 and H^Nδ^ Asn85–CO Phe4) were found in the *C*- and *N*-termini of the Lynx2 LU-domain.

In the calculated set of Lynx2 structures, large fragments of the loop I (Phe9-Asp14), loop III (Gly61-Asn72), and ‘head’ (Cys24-Gln29) were disordered (Figure 2(E2)). The calculated RMSD values (Figure 3A) demonstrated differences in plasticity of the Lynx2 loops, with the loop III being significantly less ordered than the loops I and II.

#### 2.2.4. Lynx1, SLURP-2, and Snake Toxins

Structures of the SLURP-1, Lypd6, Lypd6b, and Lynx2 LU-domains were compared with the previously published structures of human neuromodulator Lynx1 [11], human epithelial protein SLURP-2 [32], Pro33Ala mutant of ‘weak’ toxin from *Naja kaouthia* venom (WTX-P33A) [43], and short α-neurotoxin II from *Naja oxiana* venom (NTII) [44]. ^15^N relaxation data describing backbone dynamics are available for these proteins. As shown on Figure 1 and Figure 2F–I, all these proteins contain four invariant disulfide bonds in the ‘head.’ Lynx1, SLURP-2, and WTX-P33A have additional disulfide in the loop I, while short α-neurotoxin NTII does not.

Like SLURP-1 and Lynx2, these molecules involve two antiparallel β-sheets formed by five (SLURP-2, WTX, NTII) or six (Lynx1) β-strands. In the last case, residues of the loop I form three β-strands belonging to two different β-sheets (Figure 2F). There are no helices in the Lynx1, SLURP-2, WTX, and NTII molecules, but all of them (except NTII) contain disordered loops. The significant disorder (RMSD > 2 Å) was observed in the WTX loop II, Lynx1 loop III, and SLURP-2 loops II and III (Figure 3A).

### 2.3. Backbone Dynamics of TFPs in Solution

To investigate conformational plasticity of TFPs, we measured relaxation parameters of backbone ^15^N nuclei for SLURP-1, Lypd6, Lypd6b, and Lynx2, and used the published data about Lynx1, SLURP-2, WTX-P33A, and NTII [32,43,44]. To provide an unified view on the protein dynamics, the relaxation measurements were carried out under identical experimental conditions (^1^H spectrometer frequency 800 MHz and temperature 37 °C). This temperature was chosen as the closest to the physiological conditions of the human body. Dynamics of the SLURP-2 protein was previously studied at 37 °C [32]. The ‘model-free’ approach was used for analysis of ^15^N relaxation data [48]. This method permits to characterize overall rotational diffusion of the protein in solution (taking place at nanosecond timescale) and the intramolecular motions at the ps–ns and µs-ms timescales. If the µs-ms motions are in a fast regime on the NMR timescale, they lead to exchange contributions (R_EX_) to the transverse relaxation rates (R_2_), which are proportional to the square of the NMR frequency [49]. Therefore, to improve the reliability of identification of the µs-ms motions, we supplemented the ^15^N relaxation data measured at 800 MHz with R_2_ rates measured at 600 MHz. The full relaxation set (R_1_ and R_2_ rates, and ^15^N-{^1^H} NOEs) at 600 MHz was measured for Lypd6b. To diminish the contribution of the oligomerization, the relaxation data for Lynx2 were measured at lowest possible concentration (0.07 mM). For the same reason, the ^15^N relaxation data previously measured for 0.08 mM SLURP-2 [32] were used for the analysis.

It should be noted, that resonance doubling observed in the SLURP-1 loops I and II and in the *C*-terminal ‘tails’ of Lypd6 and Lypd6b is a consequence of the ms-s timescale motions, which are slow on the NMR timescale (Figure 2, column 4, dark green).

The low values of ^15^N-{^1^H} NOEs and squared generalized order parameters (S^2^, Figure S7) are the indicators of significant backbone mobility at the ps–ns timescale. On the other hand, the presence of µs-ms conformational fluctuations, which go fast on the NMR timescale, can be deduced from the increased values of R_1_ × R_2_ product [50] and presence of R_EX_ contributions (Figure S7). The ^15^N-{^1^H} NOE and R_1_ × R_2_ values depend on the NMR field strength; therefore, to define the sites of high amplitude ps–ns and µs-ms motions, we used threshold values of S^2^ (<0.8) and R_EX_ (>3.0, 2.3, and 1.7 s^−1^ at 800, 700, and 600 MHz), respectively (Figure 2, columns 3 and 4, red and blue).

The ‘model-free’ formalism assumes that S^2^ values do not depend on the magnetic field strength. For simple models of internal motions (models ##1–4) the S^2^ values provide information about amplitude of ‘fast’ ps–ns motions (typical timescale τ_e_ < 100 ps). At the same time, the more complex dynamics models (e.g., model #5) involve simultaneous ps–ns motions at two timescales: ‘fast’ (τ_f_ < 100 ps) and ‘slow’ (τ_s_~0.5–2 ns). In this case, the overall S^2^ value is equal to the product of two squared order parameters for ‘fast’ and ‘slow’ motions (S^2^ = S_f_^2^ × S_s_^2^) [49]. Therefore, the direct comparison of S^2^ values obtained for models ##1–4 and model #5 should be done with caution. However, for TFPs considered here, the model #5 was found only in a limited number of the cases; and in most of them the S_f_^2^ or S_s_^2^ or both values were <0.8 (Figure S7).

#### 2.3.1. Overall Rotational Diffusion of TFPs

Using obtained 3D structures of TFPs, we calculated their theoretical hydrodynamic properties in monomeric state at temperatures used for NMR experiments (Table 2). Weak anisotropy of the diffusion tensors (oblate, with 2D_z_/(D_x_ + D_y_) ratios in the range 1.3–1.6) and the overall rotational correlation times (τ_R_) in the range 3.7–6.1 ns (at 37 °C) were revealed. TFPs are the β-structural proteins, where the majority of HN vectors have one direction in space [51]. Therefore, we can expect that the isotropic rotational model will be sufficient to adequately represent the overall rotational diffusion of the proteins during ‘model-free’ calculations. To choose the appropriate overall rotation model, we made several runs of the ‘model-free’ analysis for each of the proteins (Table 2): one run with isotropic model and three runs with anisotropic (axially symmetric) model using different conformations of the protein from the NMR structural ensemble. The results obtained revealed very similar values of τ_R_ (Table 2) and the values of the parameters describing internal motions (e.g., S^2^ and R_EX_, see Figure S8). Thus, the use of the isotropic model did not affect the final calculation results and this model was used for the final data fits. In agreement with the orientation of HN vectors perpendicular to the direction of the highest component principal axis of the rotational diffusion tensor, the obtained experimental τ_R_ values were slightly lower than the corresponding theoretical values (Table 2). This also confirms the monomeric state of all studied TFPs at used experimental conditions (Table 1).

#### 2.3.2. SLURP-1

Both SLURP-1 isomers demonstrated similar ^15^N relaxation rates and dynamics parameters (Figure S7A). The high amplitude ps–ns motions were observed in the tips of the loops II and III, while the loop I was considerably more stable (Figure 2(A3,B3)). This agrees well with the distribution of the disordered regions in the structures (Figure 2(A2,B2)). Nevertheless, obtained mean S^2^ values revealed that the loop III is significantly more mobile than the loop II (average S^2^ is 0.66 and 0.78, respectively, Figure 3B). Thus, the observed plasticity of the loop III is connected with ps–ns motions, while disorder in the loop II probably reflects presence of both ps–ns and µs-ms timescale motions. The other sites of high amplitude ps–ns mobility were observed at the *N*- and *C*-termini and in the ‘head’ (e.g., Leu1, Cys55, and Leu81 residues). Contrary to the observed localization of ps–ns motions, the high amplitude µs-ms mobility was observed in the β-structural regions (Figure 2(A4,B4)). Probably, this is a result of propagation of conformational changes induced by *cis-trans* isomerization of the Tyr39-Pro40 peptide bond through the β-structure. This agrees well with the observed influence of *cis-trans* isomerization on the conformation of β3- and β4-strands in the loop II (see above and Figure S2A).

#### 2.3.3. Lypd6 and Lypd6b

The significant ps-ns mobility was observed in the tips of the all three loops and in the *C*-terminal ‘tail’ of Lypd6 (Figure 2(C3) and Figure S7B). The low S^2^ values in these regions agree with the high degree of structural disorder (Figure 2(C2)). In the ‘head’ of the protein only one residue with the high amplitude ps–ns mobility (Asn79) was observed. In the case of Lypd6b, the significant ps–ns mobility was observed approximately in the same regions (Figure 2(D3)), but the identification of motions in the loop II was hampered by significant broadening of ^1^H^N^ resonances (Figure 2(D3), violet). Interestingly, the ps–ns motions in the *C*-terminal ‘tail’ of the *trans-*Lypd6 isomer have larger amplitude as compared with the *cis-*isomer, as illustrated by significantly lower ^15^N-{^1^H} NOE values (Figure S7B). A direct comparison of S^2^ values in the *C*-terminal ‘tails’ was impossible. The dynamics mode of the corresponding residues is probably very complex and it has not been satisfactorily described by models ##1–5 implemented in the FastModelFree software.

Significant broadening of the HN signals of the Thr36 and His40-Ile46 residues (Lypd6 and Lypd6b, respectively) revealed the presence of µs-ms conformational fluctuations in the loop II of the proteins (Figure 2(C4,D4), violet). A comparison of R_EX_ distributions (Figure 2(C4,D4), blue) indicated that these motions have higher amplitude in the Lypd6b molecule, where corresponding conformational changes propagate through the β-sheet to the β5 strand in the loop III and α1 helix in the loop I (Figure 2(D4)). The resonance broadening in the *C*-terminal ‘tail’ of the Lypd6b *cis-*form (see above) implied that these µs-ms motions could be correlated with *cis-trans* isomerization of the Leu85-Pro86 peptide bond. Propagation of µs-ms motions in the Lypd6 molecule was restricted to the loop II residues.

#### 2.3.4. Lynx2

Similar to other TFPs studied here, the high-amplitude ps-ns mobility was observed in the loop regions of the Lynx2 molecule. Among residues with the highest ps–ns mobility are Ser17 (loop I), Glu38-Tyr45 (loop II), and Cys66 (loop III) (Figure 2(E3)). Mean S^2^ values (Figure 3B) revealed the greater mobility of the loop II at ps–ns timescale compared to the loops I and III. On the other hand, the µs-ms exchange processes were detected in almost all parts of Lynx2 (Figure 2(E4)). In particular, the significant broadening of the NMR resonances was observed in the ‘head’ (Thr25-Val28 residues) and loop III (around Ser64). Comparison of R_2_ relaxation rates of ^15^N^H^ nuclei measured at 0.25, 0.14, and 0.07 mM of Lynx2 (Figure S6D) revealed that the sample dilution resulted in the considerable decrease of R_2_ values in the loop III, ‘head,’ and β3-strand. This reflects the decrease of R_EX_ contributions to transverse relaxation. Thus, the observed µs-ms exchange processes are connected with the Lynx2 oligomerization in solution (dynamic association-dissociation). These intermolecular interactions are likely to persist even when the sample is diluted to 0.07 mM, but the population of oligomers becomes very low. Comparison of the obtained dynamics data with the results of structure calculation (Figure 2(E2)) revealed that the plasticity and disorder in the Lynx2 structure is mostly connected with µs-ms mobility.

#### 2.3.5. Lynx1, SLURP-2, and Snake Toxins

Analysis of the dynamic parameters of Lynx1, SLURP-2, WTX-P33A, and NTII (Figure 2F–I) revealed significant differences in their mobility. Similar to SLURP-1, WTX-P33A contains the regions with high-amplitude ps–ns mobility in all three loops, and µs-ms exchange fluctuations centered in the loop II (Figure 2(H34)). In spite of the significant differences in the length of the loops and secondary structure, the dynamic behavior of Lynx1 (Figure 2(F34)) resembles that observed for Lynx2. In both cases, the ps-ns mobility was observed at the periphery of the molecules, while sites of µs-ms exchange fluctuations were observed in the ‘head’ and β-structural regions of the proteins. Unlike distribution of intramolecular motions was observed in the NTII molecule (Figure 2(I34)). High-amplitude ps-ns motions were observed in the loops II and III, while µs-ms fluctuations were located mainly in the ‘head’ and loop I. Thus, similar to other TFPs the motions at different timescales are segregated in the different regions of the NTII molecule.

The dynamics mode of the SLURP-2 molecule is the most complicated and has no prototypes in other studied TFPs. All SLURP-2 loops demonstrate significant mobility both at the ps-ns and µs-ms timescales (Figure 2(G34)). The µs-ms exchange fluctuations, probably connected with SLURP-2 oligomerization observed previously [32], propagate from the loop III through the protein β-structure up to the loop I.

## 3. Discussion

nAChRs are ligand-gated ion channels and important participants of signaling in the nervous, endocrine, and immune systems of mammals [54,55]. Different nAChR subtypes are considered as promising therapeutic targets for treatment of pain, cognitive impairment, depression, and other nervous diseases [56,57,58], neuromuscular disorders [59], inflammatory and skin diseases [60], and cancer therapy [36]. Modification of endogenous ligands is one of the promising ways for design of new drugs targeted at specific receptors. Ly-6/uPAR family provides plethora of nAChR ligands. Generally, endogenous Ly-6/uPAR proteins are nAChR modulators and are active only in presence of the receptor agonist (e.g., acetylcholine). This makes them an attractive model for development of new medicines for a fine-tuning of the cholinergic system.

**Figure 4:**
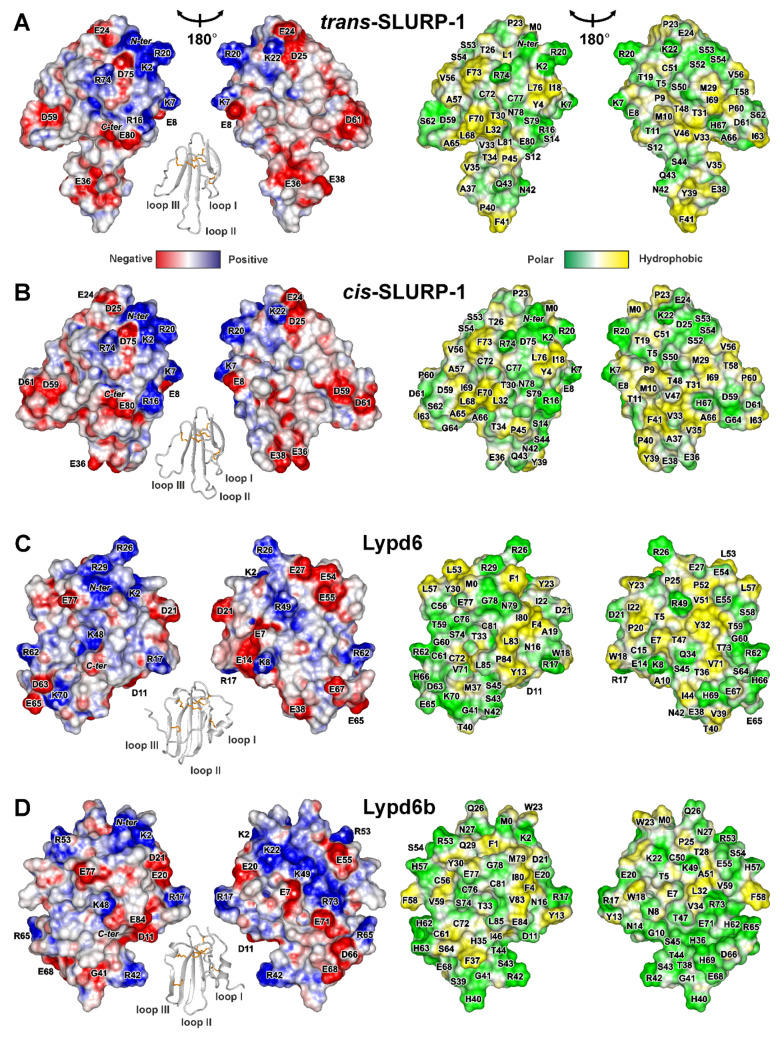

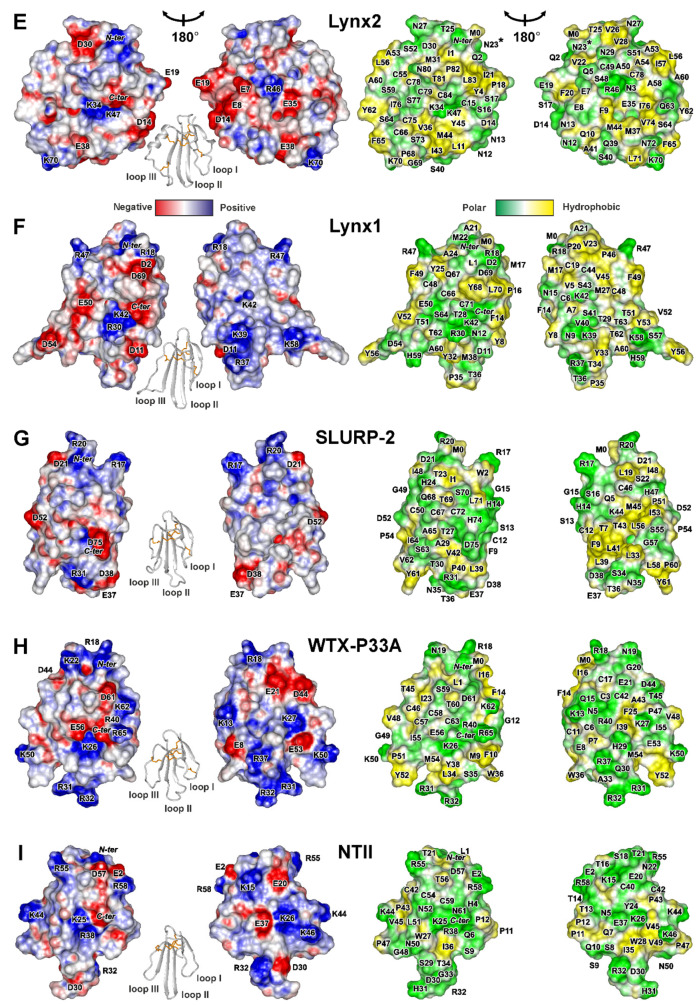
Two-sided view of molecular surfaces of TFPs: (**A**) *trans*-Tyr39-Pro40 SLURP-1, (**B**) *cis*-Tyr39-Pro40 SLURP-1, (**C**) Lypd6, (**D**) Lypd6b, (**E**) Lynx2, (**F**) Lynx1, (**G**) SLURP-2, (**H**) WTX-P33A, (**I**) NTII. Electrostatic and molecular hydrophobicity [67] potentials are shown. Red, blue, green, and yellow areas denote negative, positive, polar, and hydrophobic regions, respectively. The ribbon representation shown for each protein/isomer corresponds to the left panel. The predicted glycosylation site (Asn23) in the Lynx2 molecule is shown by asterisk.

To stimulate further drug development studies, we investigated four human Ly-6/uPAR proteins interacting with nAChRs (Lynx2, Lypd6, Lypd6b, and SLURP-1) by NMR spectroscopy. One of the advantages of NMR is an ability to determine the protein high-resolution 3D structure together with the dynamics. Dynamics and plasticity could be the important determinants of the ligand-receptor interactions. Determined structures of two SLURP-1 isomers (Figure 4A,B) illustrate how the conformational rearrangements can influence the distribution of the functional groups on the protein surface.

Positively charged and aromatic residues are required for interaction of snake three-finger α-neurotoxins with the orthosteric ligand-binding site of nAChRs [61]. Loop II of WTX, the major determinant of its interaction with the muscle and α7 neuronal receptors [62], contains three positively charged residues (Arg31, Arg32, and Arg37, Figure 4H). Similarly, the loops II and III of NTII, essential for interaction with muscle-type nAChRs, also contain several cationic groups (Lys26, Arg32, Lys44, and Lys46, Figure 4I). In contrast to that, the loops of the human TFPs do not demonstrate such high density of positive charges. For example, the prolonged loops II and III of SLURP-1, selective ligand of α7-nAChR [31], do not contain positively charged residues at all. All positively charged groups are segregated in the loop I (Figure 4A,B). This causes asymmetry in the charge distribution with one side of the SLURP-1 molecule is positively charged, while the other is negatively charged (Figure 4A,B). Notably, the SLURP-1 molecule has a significantly lower total charge (−2) compared to WTX and NTII (+6 and +4, respectively, Table 1).

Different surface properties correlate with the significant differences in activity. WTX and NTII are the nAChR antagonists that bind to the orthosteric site located between the receptor subunits [63], while SLURP-1 is a noncompetitive allosteric modulator [31] and can interact with α7-nAChR at one of the predicted allosteric binding sites [64] or at another, yet uncharacterized, site. Nevertheless, SLURP-1 shares a topology of the secondary structure with WTX and NTII and, like WTX [46,65], demonstrates conformation heterogeneity due to *cis-trans* isomerization of the Xxx-Pro peptide bond at the tip of loop II. At the same time, the SLURP-1 loops are 4–6 residues longer than those in WTX and NTII. Probably, the long, highly dynamic, and negatively charged loops determine the specific allosteric activity of SLURP-1.

Lynx1 and SLURP-2 demonstrate similarity both in the physicochemical and structural properties (Table 1, Figure 4F,G). Like SLURP-1 and WTX, these molecules have an additional fifth disulfide in the loop I and do not have α-helices in the structure. The smaller content of the positively charged residues (Arg and Lys) in the SLURP-2 molecule is compensated by larger content of His residues. Thus, at pH below 6.0, where His sidechains became positively charged, the total charge of the SLURP-2 molecule could approach the charge of Lynx1 molecule (+4) (Figure S9). It should be noted that SLURP-2 is an epithelial protein and pH values of epithelial tissues vary in wide range from 4.5 to 7.0 [47]. Unlike SLURP-1, the positively and negatively charged groups are uniformly distributed on the Lynx1 and SLURP-2 molecular surfaces, along with several hydrophobic patches (Figure 4F,G). In line with the observed structural similarity, Lynx1 and SLURP-2 demonstrate quite similar pharmacology. They inhibit α3β2- and α4β2-nAChRs, but can positively modulate the α7 receptor. Moreover, Lynx1 [11] and SLURP-2 [32], as well as WTX [66], demonstrate weak allosteric interaction with mAChRs.

Lypd6, Lypd6b, and Lynx2 have additional fifth and sixth disulfide bonds and α-helices in the loops I and III (Figure 2C–E). The helical elements are unusual among human TFPs and previously were observed only for CD59, the inhibitor of the complement membrane attack complex [5]. Topology of the β-structure in Lypd6, Lypd6b, and Lynx2 is different. Lypd6 and Lypd6b contain one wide β-sheet, formed by the residues from all three fingers of the TFP structure, while the Lynx2 β-structure involves two β-sheets and is similar to that observed in SLURPs, WTX, and NTII.

The primary structures of the Lypd6 and Lypd6b LU-domains are characterized by a relatively high similarity (60%, Figure 1). The surfaces of both proteins are relatively hydrophilic with almost uniform distribution of the differently charged and polar groups (Figure 4C,D). Nevertheless, the proteins have different physicochemical properties (Table 1). The segregation of hydrophobic/aromatic residues in the belt-like arrangement was observed for Lypd6 (Figure 4C). The ‘belt’ is composed of side-chain groups of the loop I and *C*-terminal fragment (Phe1, Phe4, Ala10, Cys15, Tyr13, Trp18, Pro20, Ile22, Tyr23, Ile80, Leu83, Pro84). These hydrophobic groups surround charged groups of Asp21 and Arg17, which could represent the potential site of the Lypd6 interaction with target receptors. Lypd6b does not contain continuous hydrophobic ‘belt’ in this region (Figure 4D), contains a relatively large number (eight) of His residues, and is less negative than Lypd6 (total charge −1 *vs* −4 at neutral pH). Difference in the surface properties implies the different pharmacology of these proteins. Indeed, the loop II motif (Asn42-Ser43-Ile44), which is responsible for the Lypd6 interaction with Wnt co-receptor LPR6 [27], is absent in Lypd6b, and, consequently, Lypd6b does not interact with LPR6 [27]. In addition, Lypd6 is able to extract the α7-nAChR subunit from the brain [23], and the loop I residues (Asp11, Tyr13, Arg17, and Trp18) probably form the site of interaction with the receptor [27]. These residues are conserved in the Lypd6b structure, but have different spatial arrangements (Figure 4C,D), which explains the lack of Lypd6b interaction with α7-nAChR [29].

The Lynx2 surface properties significantly differ from other TFPs considered in this work (Figure 4E). Segregation of the charged residues in one region formed by the loops I and II and presence of the hydrophobic patch (Ala53, Ala54, Leu56, Ile57, Ala58, Ala60, Tyr62, Phe65) on the other side of the molecule endows the protein with pronounced amphipathicity. The amphipathic properties and relative hydrophobicity (hydrophobicity index +0.07, Table 1) are probably responsible for the observed Lynx2 aggregation in solution. Interestingly, similar aggregation was previously observed for SLURP-2 [32], which also has a relatively high hydrophobicity index (+0.10, Table 1). The aggregation of Lynx2 and SLURP-2 was observed at concentrations > 0.1 mM, which are significantly higher than expected protein concentrations in the organism [15]; therefore, this aggregation, probably, has no biological consequences. One of the probable oligomerization interfaces in the Lynx2 molecule (Thr25-Val28) is located near the proposed glycosylation site (Asn23). The presence of large and polar carbohydrate substituent should protect the GPI-anchored Lynx2 molecule from aggregation. The mechanism of the Lynx2 interaction with nAChRs is currently unknown, but the obtained 3D structure suggests that the loop I with the large density of negative charges (Glu7, Glu8, Asp14, Glu19) participates in the binding to some target.

For further comparison of the TFPs structures and analysis of the similarities and differences in the architecture of the studied proteins, we performed a multiple alignment of the structures using the MAMMOTH-Mult program [68]. The data obtained (Table S5) revealed the greatest similarity between the *trans*- and *cis*-SLURP-1 isomers (96%) and between Lypd6 and Lypd6b (99%). The 3D structures of Lynx1 and SLURP-1 also showed a relatively high degree of alignment (90%). Interestingly, Lynx1, SLURP-2, and WTX constitute a cluster with a pairwise similarity exceeding 90%. This is in good agreement with a similar disulfide bonds pattern and topology of the secondary structure observed in the 3D structures. At the same time, the pair of proteins Lypd6/Lypd6b and Lynx2 do not show much similarity with other proteins and, therefore, belong to isolated structural clusters. Surprisingly, the multiple structure alignment revealed that the human CD59 protein is the most similar to Lypd6/Lypd6b (similarity > 80%).

Lynx1, Lynx2, Lypd6, and Lypd6b are the GPI-tethered molecules anchored in the membrane. Mobility and length of the membrane linker could affect the pharmacology of TFPs. Analysis of the protein sequence predicts that the GPI-anchor attachment site in the Lynx1 molecule is located immediately at the *C*-terminus of the LU-domain (Figure 1). Thus, the position and orientation of Lynx1 relative to the membrane surface cannot vary greatly, and the Lynx1 LU-domain should be oriented in the direction optimal for interaction with nAChR. In contrast to that, Lypd6 and Lypd6b have relatively long 16–19 residue linkers between the LU-domain and GPI-anchor (Figure 1). Obtained NMR data revealed that these linkers probably do not form ordered structures and demonstrate significant mobility. We can suggest that the long flexible linkers are important for Lypd6 and Lypd6b interaction with their targets, as the ligand-binding sites of the receptors can be distant from the membrane surface. Lynx2 also contains the 10-residue linker between the LU-domain and GPI-anchor. Being rich in proline and positively charged residues (Figure 1), the Lynx2 linker is probably also intrinsically disordered. The different structure of the linker regions suggests differences in the modes of Lynx1, Lynx2, and Lypd6/Lypd6b interaction with nAChRs.

NMR structures of all TFPs studied here have the disordered regions with poorly defined conformation (Figure 2). Absence of structural convergence could be a result of the structural plasticity (enhanced backbone mobility). Indeed, the qualitative comparison revealed good correspondence between the structure uncertainty and amplitude of the ps–ns timescale motions, expressed in the form of squared generalized order parameters S^2^ (Figure 3). TFP loops, which do not participate in the high-amplitude motions (S^2^ > 0.8), in general demonstrate less structural disorder (backbone RMSD < 2 Å). The interesting exception is provided by the NTII loop III, which simultaneously demonstrates the well-ordered structure and high-amplitude ps-ns dynamics (Figure 2(I3)). This clearly indicates overinterpretation of the NMR data during structure determination, which leads to artificially high precision of the resulting structure [69].

Loops II and III of the studied proteins demonstrate greater mobility at the ps–ns timescale (average S^2^ ~0.83 and 0.78, respectively) than the loop I and ‘head’ (average S^2^ ~0.86 and 0.88, respectively) (Figure 3B). Overall, SLURP-1 demonstrates the largest ps–ns mobility among the studied TFPs (average S^2^~0.80). This correlates with its 3D structure, where the β-strands of the loops II and III are significantly shorter than the overall length of the loop regions (Figure 2A,B). On the other hand, the largest overall stability (the smallest amplitude of motions) at the ps–ns timescale was observed for Lynx1, Lypd6, Lypd6b, and Lynx2 (average S^2^ 0.87–0.90, Figure 3B).

Nevertheless, the observed disorder in the TFPs structures could not be explained just by the ps–ns mobility (Figure 2). For example, disorder observed in the loop II of SLURP-1 and WTX-P33A probably also reflects the presence of the µs-ms timescale motions. Indeed, the exchange contribution to the R_2_ relaxation rates was observed in all proteins studied (Figure 2). Conformational exchange processes in SLURP-1, Lypd6, and Lypd6b could be due to *cis-trans* isomerization of the Xxx-Pro peptide bonds, while in Lynx2 and SLURP-2 the exchange processes can be related to the weak protein oligomerization in solution. Interestingly, wild type WTX demonstrates conformation heterogeneity in solution due to *cis-trans* isomerization of the Arg32-Pro33 peptide bond located in the loop II; and this dynamic process, similarly to the situation with SLURP-1, influences the loop I conformation [43,65]. WTX-P33A mutant lacks the corresponding Pro residue and, therefore, adopts only one isomeric state in solution. Nevertheless, the µs-ms timescale motions are still present in the loop II and in the adjacent regions of the molecule (Figure 2(H4)). This paradoxical situation implies that the conformational plasticity (a tendency to fluctuations) is an inherent property of the WTX loop II, and the Pro residue provides just an outlet for this tendency, concentrating it in the exchange between two isomeric forms.

What is the biological meaning of the plasticity of TFPs structures? We can propose several hypotheses:

(1) The ligand plasticity is required to adopt its structure for interaction with several molecular targets. Indeed, many of the studied proteins (WTX, Lynx1, Lypd6, and SLURP-2) can interact with nAChRs and other receptors, e.g., mAChRs or LPR6. In this case, the high plasticity of SLURP-1 suggests the presence of a molecular target other than α7-nAChR. Currently, this putative target remains uncharacterized.
(2) The ligand plasticity is needed for adaptation to the plasticity of the nAChR binding sites. The binding sites of the receptor experience significant rearrangements during gating of the receptor ion channel [70]. Indeed, the endogenous human TFPs are not direct blockers or agonists of nAChR, but rather are modulators of the receptor [1]. They probably bind the receptor in different conformational states and do not prevent the movements of the receptor during gating, but rather influence populations of different states. The ability of human TFPs to interact with different functional states of nAChR is supported by the ability of Lynx1 and SLURP-2 to enhance or suppress ion currents through α7-nAChR [11,32].
(3) The ligand plasticity is a mechanism, which regulates the affinity of ligand-receptor interaction via entropic term of the free energy (*ΔG = ΔH − TΔS*). Indeed, the binding of WTX-P33A to human M1 mAChR results in the significant decrease of the loops I and II mobility at the ps–ns timescale [43]. This causes a loss of conformational entropy, which makes formation of the ligand-receptor complex less energetically favorable. The simple calculations permit to estimate the magnitude of this effect [71]. An increase in the average S^2^ value for a 15-residue polypeptide from 0.7 to 0.85 upon the receptor binding leads to entropic penalty (*−TΔS*) ~+3.5 kcal/mol (~5.7·k_B_·T) at 30 °C (only change in backbone conformational entropy was taken into account). According to the Boltzmann equation, such *ΔG* difference can lead to the more than two order of magnitude difference in affinity (dissociation constant) between the ‘mobile’ and ‘stable’ protein ligands. In real situations, the unfavorable conformational entropy term is probably compensated by the favorable enthalpy and entropy terms due to better match between the ligand and receptor structures. For example, WTX and NTII have similar overall length (65 and 61 residues) and very close average S^2^ values (0.84 and 0.85, respectively). The larger mobility of the NTII loop III is compensated by larger mobility of the WTX loop I (Figure 3B). Despite this similarity, the affinity of toxins for muscle-type nAChRs is significantly different (IC_50_~3 µM and 30 nM for WTX and NTII, respectively) [62,72], indicating the involvement of structural factors other than intramolecular mobility. Nevertheless, the above calculations indicate that the change in conformational entropy of interacting molecules upon the complex formation cannot be neglected.

We suppose that all the above hypotheses are operative, and conformational plasticity is an important factor in the successful interaction of three-finger ligands with nAChRs and other targets and is an inherent property of TFPs. Moreover, the plasticity is not restricted to the loops rich in the ps–ns motions, but also is observed in the β-structural core, where µs-ms fluctuations freely propagate between neighboring β-strands.

## 4. Materials and Methods

### 4.1. Recombinant Production and Samples Preparation

SLURP-1, Lypd6, Lypd6b, and Lynx2, and their isotopically ^15^N- and ^13^C,^15^N-labeled variants, were expressed in the form of *E. coli* inclusion bodies. The unfolded proteins were solubilized and refolded using general protocol developed for TFPs production [41]. The details about the protocol optimization for the individual proteins are given in previous publications [42,46,48]. Refolding of the proteins was controlled by 1D ^1^H NMR spectroscopy, assuming that a correctly folded TFP should contain a significant amount of the β-structure and demonstrate high dispersion of signals in HN and methyl regions of the NMR spectrum. Purification of the refolded proteins was performed by HPLC using Jupiter C4 column (A300, 4.6 × 250 mm, Phenomenex, Torrance, CA, USA). The purity and homogeneity of the refolded proteins were confirmed by HPLC, MALDI-MS, and SDS-PAGE. Disulfide bond formation was confirmed using Ellman’s reagent.

NMR samples were prepared by dissolving of the lyophilized proteins in the 0.3 mL of mQ (Millipore) water. D_2_O (5%) was added to each sample, and pH was adjusted by adding small volumes of concentrated HCl or NaOH. Samples were placed into Shigemi NMR tubes.

### 4.2. NMR Spectroscopy

The samples of ^13^C,^15^N- and ^15^N-labeled proteins were used for NMR study. Experimental conditions are shown in Table 1. NMR spectra were measured at AVANCE-III-600 and AVANCE-III-800 spectrometers (Bruker, Germany) equipped with CryoProbes. 3D spectra were acquired using non-uniform sampling method with 30% of sparse sampling for triple-resonance and 50% for 3D TOCSY-HSQC and NOESY-HSQC spectra, and processed with MDDNMR [73]. The backbone resonance assignment was performed using standard set of 3D triple-resonance (^1^H,^13^C,^15^N) NMR experiments (HNCO, HNCA, HNCACB, HN(CO)CA, HN(CO)CACB, and HN(CA)CO [74]). 3D ^13^C-HCCH-TOCSY [74] and ^15^N- or ^13^C-filtered 3D TOCSY-HSQC and NOESY-HSQC spectra [75] were used for side chains assignment. ^3^J_H_^N^_H_^α^ scalar coupling constants were measured using 3D HNHA spectrum [76]. ^3^J_H_^β^_N_ couplings were estimated from cross peak intensities in 3D HNHB spectrum [76]. Temperature gradients of amide protons (Δδ^1^H^N^/ΔT) were extracted from series of ^15^N-HSQC spectra measured in the 20–45 °C temperature range. To identify slowly exchanging amide protons, ^15^N-labeled variants of SLURP-1 and Lypd6 were dissolved in 100% D_2_O. The H/D exchange kinetics was measured using ^15^N-HSQC spectra.

Relaxation parameters of ^15^N nuclei (longitudinal (R_1_) and transverse (R_2_) relaxation rates and steady-state heteronuclear ^15^N-{^1^H} NOEs) for the SLURP-1, Lypd6, Lypd6b, and Lynx2 proteins were measured at frequencies and temperatures listed in Table 1. Temperature calibration for accurate matching between the two spectrometers was performed using a sample of pure methanol-d4 (99.9% of ^2^H) by measuring chemical shift difference between the residual ^1^H signals of methyl and hydroxyl groups [77]. Relaxation measurements were done using a standard set of ^15^N-HSQC-based pseudo 3D experiments [49]. For ^15^N-{^1^H}-NOE measurements, ^1^H nuclei were presaturated by a sequence of 120° pulses over 4 s. Eight relaxation delays of 100, 200, 300, 500, 800, 1000, 1200, and 1500 ms were used for R_1_ measurements. To estimate experimental uncertainty, the additional 2D plans with 300 and 800 ms delays were measured. Eight delays with CPMG sequence length of 17, 34, 51, 68, 85, 102, 136, and 170 ms were used for R_2_ measurements. To estimate experimental uncertainty, the additional 2D plans with 34 and 136 ms CPMG sequence lengths were measured. The values of ^15^N relaxation parameters for Lynx1, SLURP-2, WTX, and NTII were taken from the previous reports [11,32,43,44] and from the Biological Magnetic Resonance Data Bank (BMRB ID 19704 for NTII).

### 4.3. Structure Calculation and Relaxation Data Analysis

Secondary structure of SLURP-1, Lypd6, Lypd6b, and Lynx2 was calculated from ^1^H, ^13^C, and ^15^N chemical shifts using TALOS-N [78]. For 3D structure calculation, distance constraints were derived from cross peak intensities in ^15^N- or ^13^C-filtered 3D NOESY-HSQC spectra (τ_m_ = 100–120 ms). The φ and χ_1_ dihedral angles restraints were obtained from J-couplings, NOE, and TALOS data. The hydrogen bonding restraints were applied, assuming that an amide proton with Δδ^1^H^N^/ΔT > −4.5 ppb/K or having H/D half-exchange time > 20 min could participate in the hydrogen bond formation. Additional distance restraints were applied to restrain disulfide connectivity. 3D structures were calculated using CYANA ver. 3.98 [79].

Visualization and analysis of the calculated structures were performed using MOLMOL [80]. Initially the sets of 20 resulting structures for each of the proteins were superimposed using coordinates of backbone atoms (C^α^, C’, N) in the ‘stable’ (not disordered) protein regions (Table S2). Loop regions were defined relative to positions of the conservative Cys residues (Figure 1, gray shades). Mean RMSD values for backbone atoms in the loops, *C*-terminal regions of Lypd6 and Lypd6b (residues Pro86-Ala95, Figure 1, gray shade), and in the ‘heads’ of the proteins (other residues excluding first one) were calculated. The structures of Lynx1, SLURP-2, WTX-P33A, and NTII were taken from PDB, accession codes 2L03, 2N99, 2MJ0, and 2MJ4, respectively. Multiple alignment of 3D protein structures was performed using the MAMMOTH-Mult program [68].

Relaxation data were analyzed using FastModelFree [53]. Isotropic or anisotropic (with the oblate axially symmetric tensor) diffusion models were used. Mean S^2^ values were calculated for the same protein regions, which were used for RMSD calculations. Hydrodynamic calculations were performed in the HYDRONMR software [52].

### 4.4. Accessing Codes

Experimental restraints and atomic coordinates for the *trans*-SLURP-1, *cis*-SLURP-1, *trans*-Lypd6, *trans*-Lypd6b, and Lynx2 structures have been deposited in PDB under accession codes 6ZZE, 6ZZF, 6IB6, 6ZSO, and 6ZSS, respectively. Chemical shifts, and relaxation data were deposited in BMRB under accession codes 34547, 34548, 34333, 34531, and 34532, respectively.

## 5. Conclusions

Two different topologies of the β-structure were observed in the LU-domains of human TFPs. Large antiparallel five-stranded β-sheet is present in the Lypd6 and Lypd6b molecules, while other proteins have two β-sheets composed of six (Lynx1) or five (SLURP-1, SLURP-2, and Lynx2) β-strands. α-Helical segments were observed only in the loops I and III of Lynx2, Lypd6, and Lypd6b.

Comparison of the TFPs structures did not reveal any conserved pattern of charged/hydrophobic/aromatic groups. This indicates substantial differences in the mode of interaction with nAChRs or in the specificity to different receptor subtypes.

The linkers between LU-domain and GPI-anchor of Lynx1, Lynx2, Lypd6, and Lypd6b have different lengths. The shortest and, probably, most rigid two-residue linker is present in the Lynx1 molecule, while the longest and very mobile 16–19 residue linkers are in Lypd6 and Lypd6b. The 10-residue Lynx2 linker is, probably, also highly mobile. The properties of the linker region could influence the pharmacology of TFPs.

The ms–s timescale conformational fluctuations due to *cis-trans* isomerization of the Xxx-Pro peptide bonds were observed in the loop II of SLURP-1 and in the *C*-termini of Lypd6 and Lypd6b.

Human TFPs and snake toxins demonstrate high conformational plasticity. The ps–ns timescale motions were observed in the loops, while the main sites of the µs-ms fluctuations are located in the β-structural core. The loops II and III demonstrate greater mobility at the ps–ns timescale as compared to the loop I and ‘head.’ Overall, SLURP-1 demonstrates the largest amplitude of the ps–ns motions. Conformational plasticity may be an important feature of TFPs, providing adaptation of their structures for binding to the different receptors in different conformational states, and allowing control of the free energy of ligand-receptor complex formation.

The obtained data revealed complex relationships between the structure, dynamics, and function of the Ly6/uPAR proteins acting on nAChRs. The knowledge of the structure-dynamic landscape of the endogenous three-finger molecules is needed for rational drug design by computational methods.

## Figures and Tables

**Table 1:** Physicochemical properties of TFPs and experimental conditions for NMR measurements.

Protein	pI	Charges (+/−/His) ^a^	HI ^b^	Labeling	Conc., mM	pH	T, °C	Freq., MHz ^c^	PDB	Ref
SLURP-1	5.2	8/10/1	0.04	^13^C, ^15^N	0.3	4.7	37	800	6ZZE 6ZZF	This work
Lypd6	5.3	11/15/3	−0.58	^13^C, ^15^N/^15^N ^d^	0.1	7.0	30/37 ^d^	600/800	6IB6	This work
Lypd6b	6.7	10/11/8	−0.68	^13^C, ^15^N	0.2	5.5	30/37 ^d^	600/800	6ZSO	This work
Lynx2	4.5	5/8/0	0.07	^13^C, ^15^N	0.25/0.07 ^d^	6.7	45/37 ^d^	600/800	6ZSS	This work
Lynx1	8.1	8/6/2	−0.32	^15^N	0.5	5.3	25	800	2L03	[11]
SLURP-2	6.5	5/6/5	0.10	^13^C, ^15^N	0.5 ^e^/0.08 ^d^	4.8	37	600	2N99	[32]
WTX-P33A	8.9	13/7/1	−0.39	^15^N	0.5	3.0	30	700	2MJ0	[43]
NTII	8.7	10/6/2	−1.10	^15^N	1.0	5.0	30	800	2MJ4	[44]

^a^ The number of positively (“+”, Arg/Lys/*N*-terminus) and negatively (“−“, Asp/Glu/*C*-terminus) charged groups in the protein at neutral pH. The number of His residues, which could change their ionization state at a pH of about 6.0, is also shown. ^b^ Mean Kyte-Doolittle hydrophobicity index [45]. The maximum and minimum values of this index are +4.5 and −4.5 for poly-Ile and poly-Arg sequences, respectively. ^c 1^H frequency of the NMR spectrometer, where the ^15^N relaxation was measured. ^d^ First value shows the sample, its concentration, or temperature used for structure calculation; the second shows the same value for the ^15^N relaxation measurements. ^e^ 5% dioxane was added to the sample to prevent aggregation.

**Table 2:** Rotational diffusion of TFPs in solution.

Protein	T, °C	Theoretical ^a^			Experimental ^b^	
		τ_R_, ns	Anisotropy 2D_z_/(D_x_ + D_y_)	Asymmetry D_x_/D_y_	τ_R_, ns (iso/axial)	Anisotropy 2D_z_/(D_x_ + D_y_)
*trans*-SLURP-1	37	5.0	1.56	1.06	4.3/4.9	1.50
*cis*-SLURP-1	37	5.1	1.44	1.11	4.3/4.8	1.78
Lypd6	37	6.1	1.30	1.10	4.6/4.6	1.26
Lypd6b	37	6.1	1.36	1.11	5.0/5.0	1.32
Lynx2	37	3.7	1.25	1.17	4.0/3.8	1.35
Lynx1	25	6.1	1.56	1.14	5.0/5.2	1.57
SLURP-2	37	4.0	1.60	1.01	4.0/4.1	1.42
WTX-P33A	30	3.7	1.49	1.06	3.6/3.6	1.34
NTII	30	3.7	1.50	1.08	2.9/3.0	1.42

^a^ The values were calculated using the three-dimensional (3D) structures of proteins and the HYDRONMR program [52]. ^b^ The values were calculated in the FastModelFree program [53] using the ^15^N relaxation data assuming isotropic or axially symmetric rotational diffusion tensor. The values shown for axially symmetric rotational model are the average of three independent calculations using different protein structures.

## Supplementary Materials

Supplementary materials can be found at https://www.mdpi.com/1422-0067/21/19/7280/s1. Table S1: Extent of NMR resonance assignment for SLURP-1, Lypd6, Lypd6b, and Lynx2; Table S2: Loop regions for the RMSD and mean S^2^ calculations. Residue ranges are given. The backbone atoms in the ordered regions were used to superimpose structures before RMSD calculation; Table S3: Selected distances between the atoms of the Lypd6 *C*-terminal fragment (Pro85-Ala95) and the atoms of the Lypd6 molecule from the neighboring asymmetric unit of the crystal lattice. (PDB 6GBI); Table S4: Statistics for the best CYANA structures of SLURP-1, Lypd6, Lypd6b, and Lynx2; Table S5: Pairwise similarity of 3D structures of the TFPs; Figure S1: Temperature dependence of ^1^H^N^ chemical shifts of SLURP-1 (A), Lypd6 (B), Lypd6b (C), and Lynx2 (D); Figure S2: NMR data define the secondary structure and conformational heterogeneity of SLURP-1 (A), Lypd6 (B), Lypd6b (C), and Lynx2 (D); Figure S3: ^13^C-HSQC spectrum of ^13^C,^15^N-labelled SLURP-1 illustrate conformational heterogeneity and Pro40 assignment; Figure S4: Comparison of the Lypd6 3D structures obtained by X-ray (Red, PDB 6GBI) and NMR (Grey, PDB 6IB6); Figure S5: Contacts between neighboring asymmetric units in the crystal structure of Lypd6 (PDB 6GBI); Figure S6: NMR spectral parameters of Lynx2 at different concentrations and temperature; Figure S7: ^15^N relaxation data and results of the ‘model-free’ analysis for SLURP-1 (A), Lypd6 (B), Lypd6b (C), Lynx2 (D), Lynx1 (E), SLURP-2 (F), WTX-P33A (G), and NTII (H); Figure S8: Choice of overall rotational diffusion model for the ‘model-free’ analysis of ^15^N relaxation data for SLURP-1 (A1, A2), Lypd6 (B), Lypd6b (C), Lynx2 (D), Lynx1 (E), SLURP-2 (F), WTX-P33A (G), and NTII (H); Figure S9: Two-sided view of electrostatic potential on the SLURP-2 molecular surface at weakly acidic pH.
