# Supplementary material for: Structural Diversity and Dynamics of Human Three-Finger Proteins Acting on Nicotinic Acetylcholine Receptors
Source: Int J Mol Sci. 2020 Oct 1;21(19):7280. doi: 10.3390/ijms21197280 (PMC7582953; doi:10.3390/ijms21197280)
Supplement: Supplementary file 1 [file ijms-21-07280-s001.pdf]

International Journal of
Molecular Sciences

MDPI

Supplementary

Structural Diversity and Dynamics of Human Three-Finger Proteins Acting on Nicotinic Acetylcholine Receptors

Alexander S. Paramonov [1], Milita V. Kocharovskaya [1,2], Andrey V. Tsarev [1,2], Dmitrii S. Kulbatskii [1], Eugene V. Loktyushov [1], Mikhail A. Shulepko [1], Mikhail P. Kirpichnikov [1,3], Ekaterina N. Lyukmanova [1,2,*] and Zakhar O. Shenkarev [1,2,*]

1 Shemyakin-Ovchinnikov Institute of Bioorganic Chemistry, Russian Academy of Sciences, 119997 Moscow, Russia; a.s.paramonov@gmail.com (A.S.P.); kocharovskaya.mv@phystech.edu (M.V.K.); tsarev2709@yandex.ru (A.V.T.); d.kulbatskiy@gmail.com (D.S.K.); zhenyaloktushov@mail.ru (E.V.L.); mikhailshulepko@gmail.com (M.A.S.); kirpichnikov@inbox.ru (M.P.K.)
2 Phystech School of Biological and Medical Physics, Moscow Institute of Physics and Technology (National Research University), 141701 Dolgoprudny, Moscow Region, Russia
3 Faculty of Biology, Lomonosov Moscow State University, 119234 Moscow, Russia
* Correspondence: ekaterina-lyukmanova@yandex.ru (E.N.L.); zakhar-shenkarev@yandex.ru (Z.O.S.)

Received: 7 August 2020; Accepted: 28 September 2020; Published: date

Supplementary Tables

Table 1. Extent of NMR resonance assignment for SLURP-1, Lypd6, Lypd6b, and Lynx2.

	**SLURP-1 'trans-' ('cis-')**		**Lypd6**		**Lypd6b**		**Lynx2**	
	**Assigned atoms**	**%**	**Assigned atoms**	**%**	**Assigned atoms**	**%**	**Assigned atoms**	**%**
**H^N**	76 (76)	98 (98%)	88	97%	90	95%	75	90%
**$H^α/C^α$**	83/80 (83/82)	100/98% (100/100%)	99/95	99/100%	99/95	99/100%	84/83	97/97%
**$H^β/C^β$**	150/78 (150/81)	100/96% (100/100%)	170/91	98/99%	162/91	93/99%	146/78	91/94%
**C'**	63 (72)	77% (87%)	87	90%	87	90%	74	86%
**H aliphatic** (except α and β)	205 (207)	74% (75%)	243	76%	178	58%	187	71%
**H aromatic**	15 (19)	60% (76%)	36	97%	45	100%	16	53%

Table 2. Loop regions for the RMSD and mean S^2 calculations. Residue ranges are given. The backbone atoms in the ordered regions were used to superimpose structures before RMSD calculation.

Protein	loop I	loop II	loop III	Ordered regions
SLURP-1 (both isomers)	6-18	32-47	58-68	1-6,15-32,47-57,68-78
Lypd6	6-21	35-46	59-72	6-8,30-37,44-52,69-75
Lypd6b	6-21	35-46	59-72	2-6,10-18,22-33,47-57,73-84
Lynx2	6-21	36-45	58-75	2-7,20-23,31-36,44-56,75-85
Lynx1	6-16	30-40	51-62	1-46,62-72
SLURP-2	6-15	29-42	53-63	1-7,13-29,42-46,67-72
WTX P33A	6-14	28-38	49-54	1-6,12-28,38-63
NTII	6-14	27-36	45-50	1-61

Table 3. Selected distances between the atoms of the Lypd6 *C*-terminal fragment (Pro85-Ala95) and the atoms of the Lypd6 molecule from the neighboring asymmetric unit of the crystal lattice. (PDB 6GBI).

Atoms	Distance, Å
Glu89 Hγ - Glu89 Hγ	2.60
Phe94 Hε - Leu53 Hδ	2.62
Ala95 Hα – Leu57 Hβ	2.16

Table 4. Statistics for the best CYANA structures of SLURP-1, Lypd6, Lypd6b, and Lynx2.

**Protein**	**SLURP-1**		**Lypd6**	**Lypd6b**	**Lynx2**
**Experimental conditions**	pH 3.4, 37 ºC		pH 7.0, 30 ºC	pH 5.5, 30 ºC	pH 6.7, 45 ºC
**Distance and Angle restraints**	*trans*-Y39-P40	*cis*-Y39-P40			
Total NOE contacts	493	504	467	686	522
intraresidual	176	179	210	157	150
sequential (|i-j|=1)	182	184	171	254	195
medium-range (1<|i-j|≤4)	21	23	14	70	42
long-range (|i-j|>4)	114	118	72	205	135
Hydrogen bonds restraints (upper/lower)	42/42	42/42	64/64	76/76	66/66
S-S bond restraints (upper/lower)	15/15	15/15	18/18	18/18	18/18
Torsion angle restraints	137	144	103	134	83
Angle φ	63	70	67	71	56
Angle $\chi1$	74	74	36	63	27
**Total restraints/per residue:**	740/9.1	758/9.4	734/7.6	1008/10.5	773/9.0
**Statistics for calculated structures**					
Structures calculated/selected	200/20	200/20	500/20	200/20	200/20
CYANA target function (Å^2)	2.10 ± 0.14	2.61 ± 0.19	2.02 ± 0.17	2.25 ± 0.37	2.53 ± 0.36
Violations of restraints					
Distance (>0.2 Å)	6	6	4	3	5
Distance (>0.5 Å)	0	0	0	0	0
Dihedral angles (>5 °)	0	0	0	1	0
**RMSD (Å)**					
Overall					
Backbone	2.85± 0.77	2.47 ± 0.58	2.44 ± 0.61	4.74 ± 1.98	1.76 ± 0.35
Heavy atoms	3.66± 0.76	3.25 ± 0.53	3.26 ± 0.49	5.09 ± 1.83	2.39 ± 0.35
Ordered regions (see Table S1)					
Backbone	0.33 ± 0.06	0.34 ± 0.06	0.55 ± 0.13	0.47 ± 0.10	0.71 ± 0.13
Heavy atoms	1.08 ± 0.16	1.10 ± 0.16	1.42 ± 0.17	1.26 ± 0.13	1.41 ± 0.15
**Ramachandran analysis (backbone)**					
Residues in favored regions (%)	89	92	86	86	83
Residues in favored and allowed regions (%)	97	98	99	99	99

Table 5. Pairwise similarity of 3D structures of the TFPs.

	tSLURP1	cSLURP1	LYPD6	LYPD6B	LYNX2	LYNX1	SLURP2	WTX	NTII	α-Cbtx	Mbl-1	Ctx-1	CD59
**t-SLURP1**	100	96	66	78	58	90	85	78	85	81	73	81	73
**c-SLURP1**	96	100	59	72	71	90	83	88	85	79	80	88	70
**LYPD6**	66	59	100	99	76	73	80	77	82	26	75	71	82
**LYPD6B**	78	72	99	100	85	88	83	89	28	26	75	71	87
**LYNX2**	58	71	76	85	100	85	76	80	78	59	68	73	66
**LYNX1**	90	90	73	88	85	100	95	92	88	71	88	86	79
**SLURP2**	85	83	80	83	76	95	100	94	93	81	82	75	75
**WTX**	78	88	77	89	80	92	94	100	95	80	93	100	78
**NTII**	85	85	82	28	78	88	93	95	100	82	91	92	70
**α-cobratoxin**	81	79	26	26	59	71	81	80	82	100	84	97	66
**Mbl-1**	73	80	75	75	68	88	82	93	91	84	100	84	64
**Cytotoxin-1**	81	88	71	71	73	86	75	100	92	97	84	100	66
**CD59**	73	70	82	87	66	79	75	78	70	66	64	66	100

The similarities were calculated using the MAMMOTH-Mult program [*Lupyan D, Leo-Macias A, Ortiz AR (2005) Bioinformatics (2005) 21, 3255-63*]. The pairs with similarity >90% are marked by green background. Data for the proteins studied in this work are shown on a gray background.

PDB codes: α-cobratoxin – 2CTX, neurotoxin II – 2MJ4, mambalgin-1 – 5DU1, cytotoxin I – 5NPN, WTX – 2MJ0, SLURP-1 – 6ZZE/6ZZF, SLURP-2 – 2N99, CD59 – 2J8B, Lynx1 – 2L03, Lynx2 – 6ZSS, Lypd6 – 6IB6, Lypd6B – 6ZSO.

Supplementary Figures

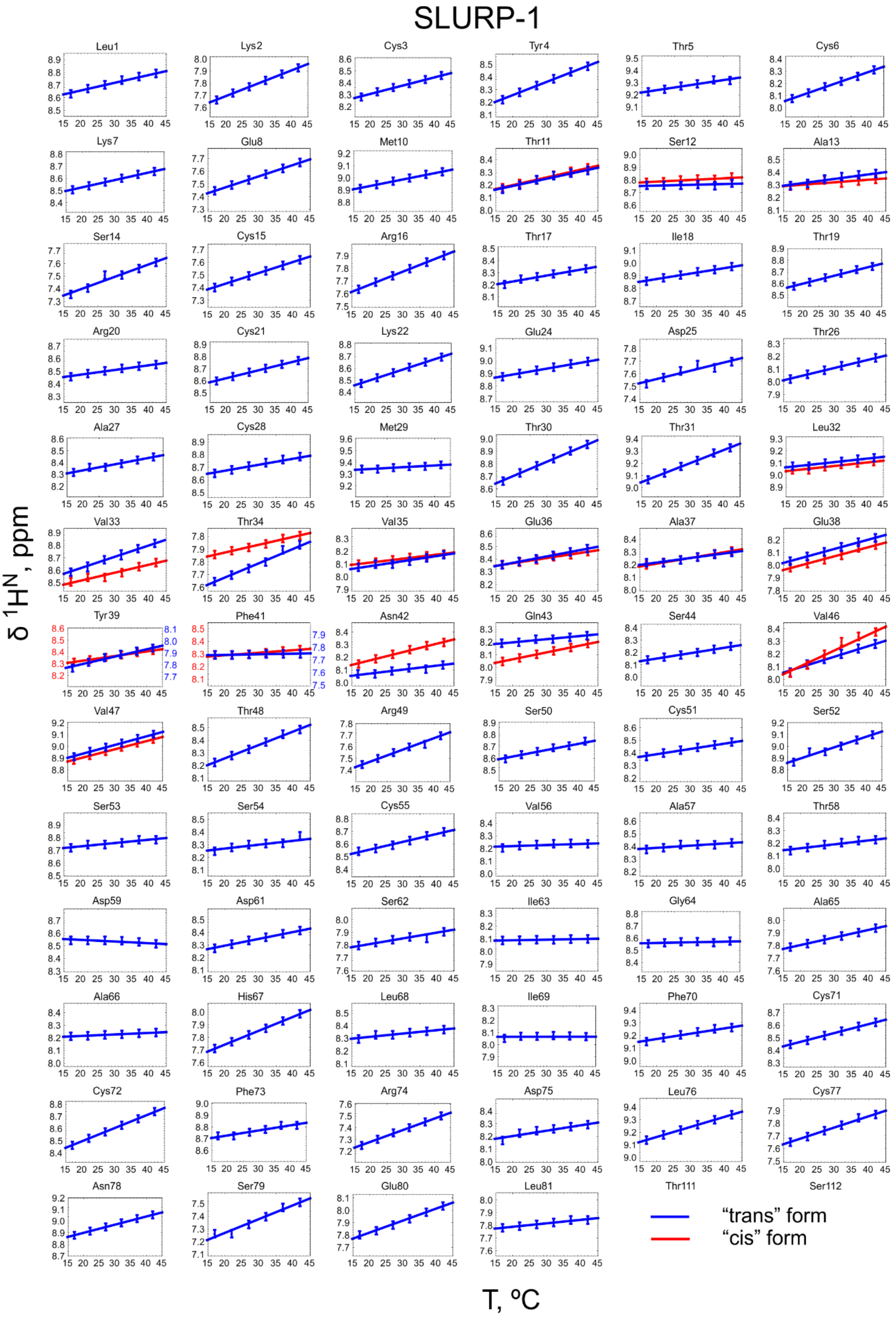

Figure 1A. Temperature dependence of $^1H^N$ chemical shifts of SLURP-1. The data for *cis*-isomer are shown by red symbols.

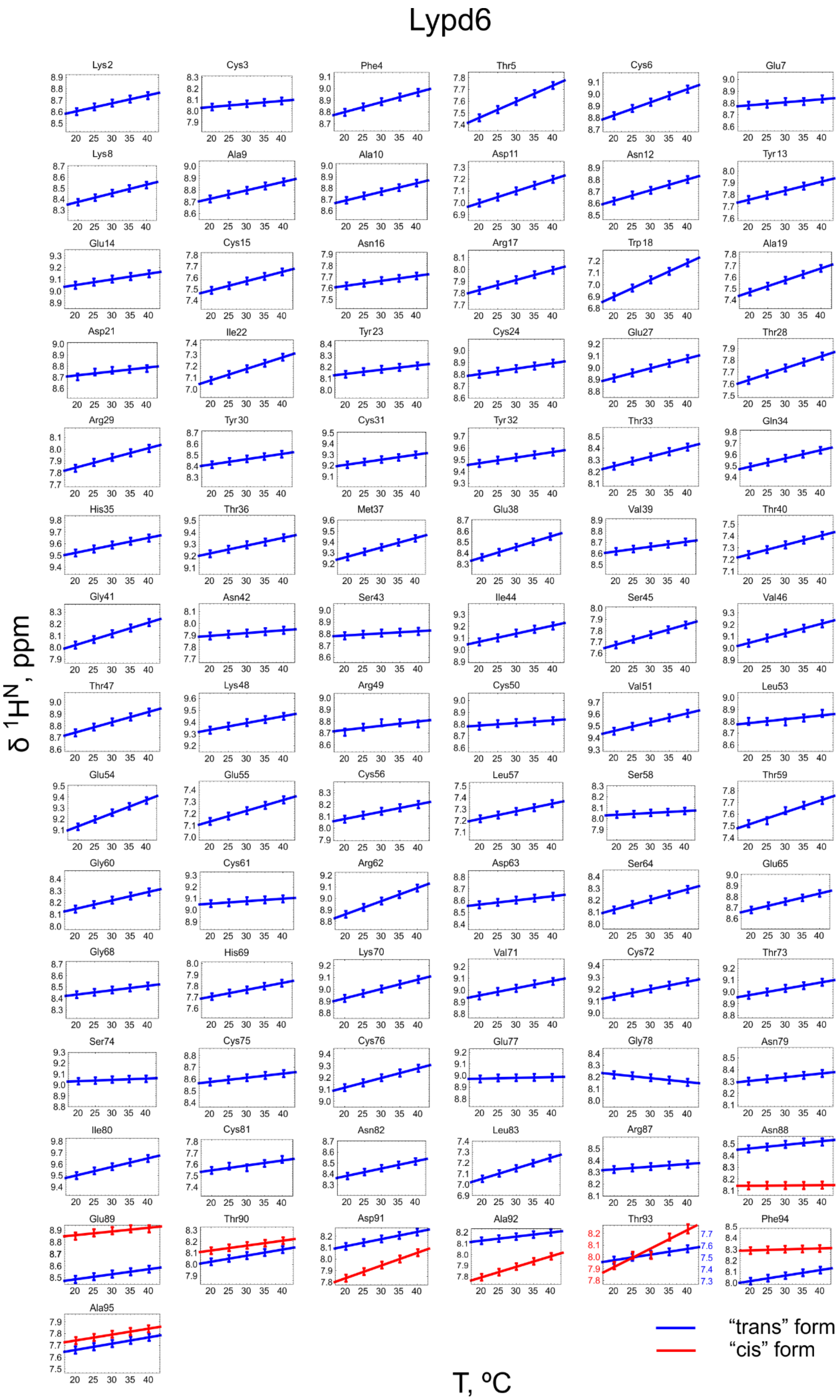

Figure 1B. Temperature dependence of $^{1}H^{N}$ chemical shifts of Lypd6. The data for *cis*-isomer are shown by red symbols.

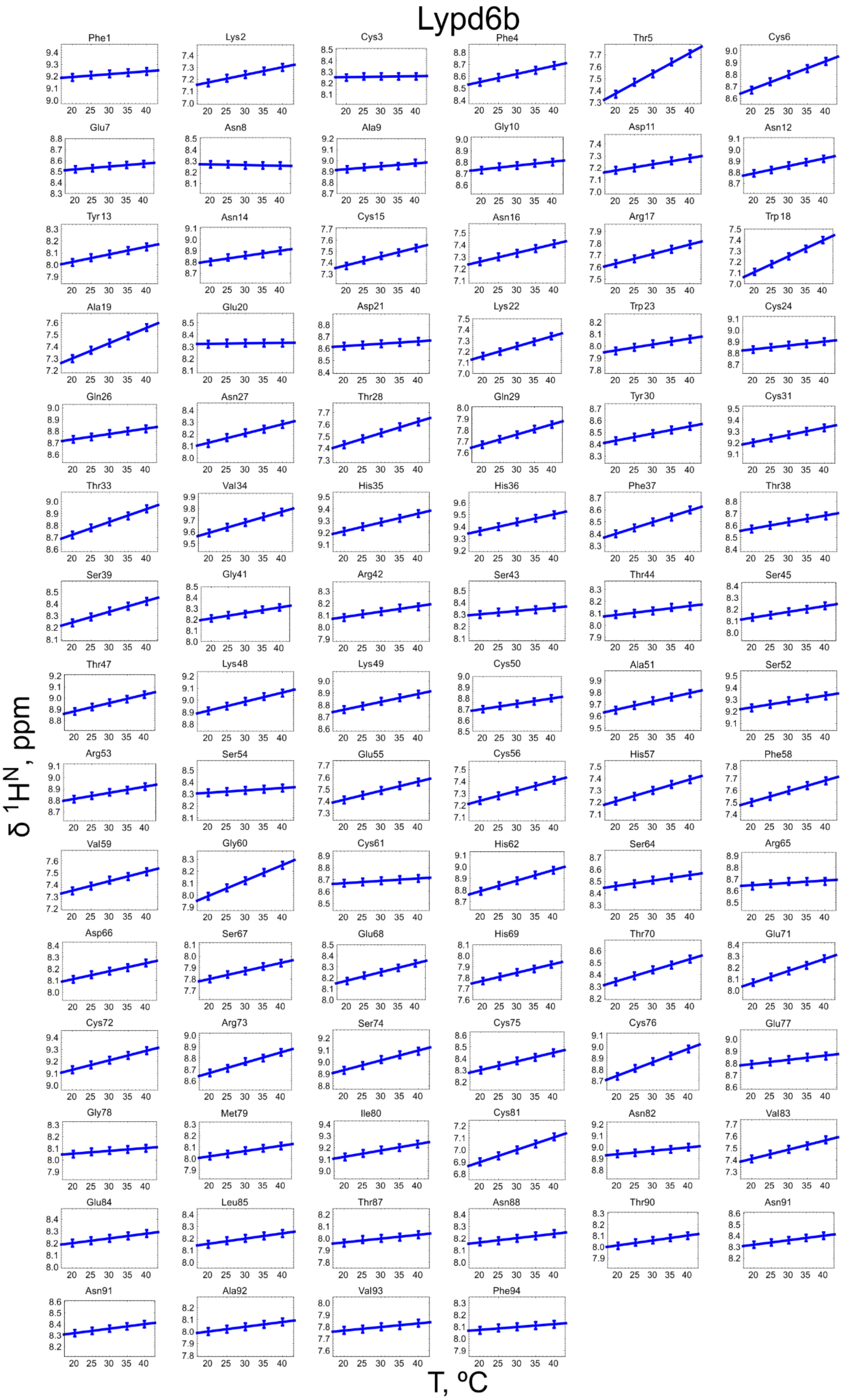

Figure 1C. Temperature dependence of $^{1}H^{N}$ chemical shifts of *trans*-Lypd6.

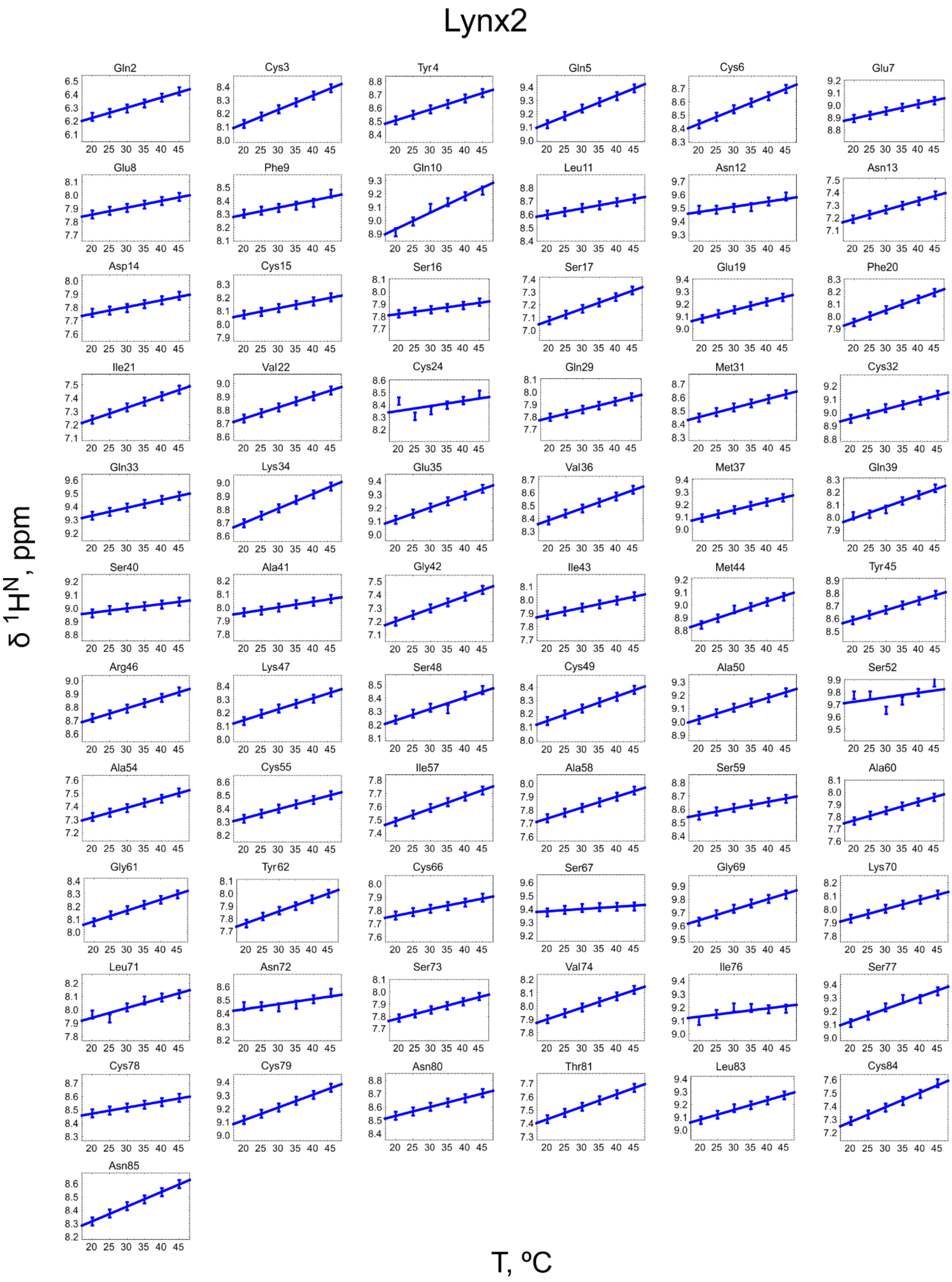

Figure 1D. Temperature dependence of $^{1}H^{N}$ chemical shifts of Lynx2.

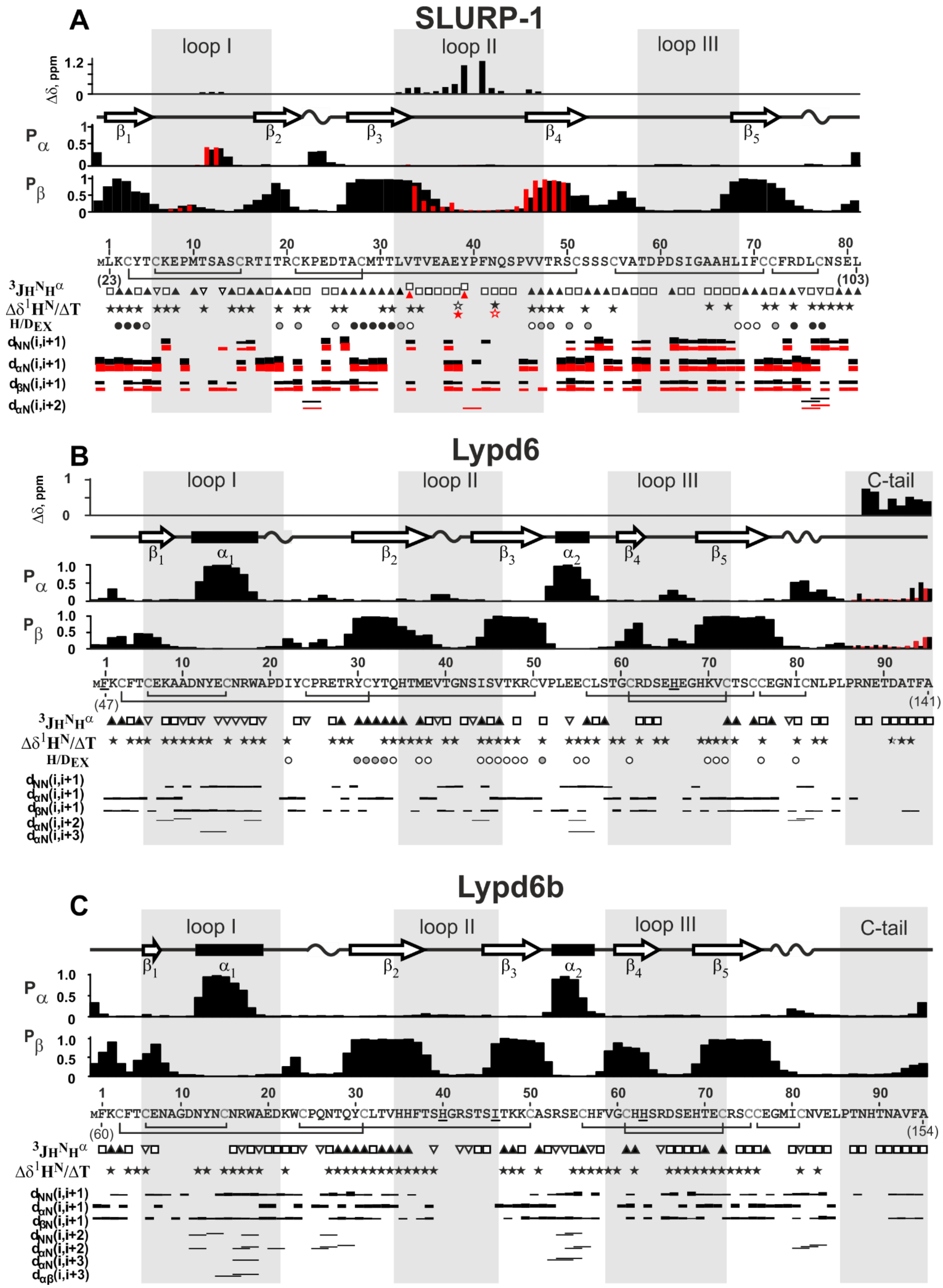

A
SLURP-1
loop I
loop II
loop III
Δδ, ppm
Pα
Pβ
MLKCYTCKEPMTSASCRTITRCKPEDTACMTTLVTVEAEYPFNQSPVVTRSCSSSCVATDPDSIGAAHLIFCCFRDLCNSEL
(23)
(103)
³JHNHα
Δδ¹HN/ΔT
H/DEX
dNN(i,i+1)
dαN(i,i+1)
dβN(i,i+1)
dαN(i,i+2)
B
Lypd6
C-tail
MFKCFTCEKAADNYECNRWAPDIYCPRETRYCYTQHTMEVTGNSISVTKRCVPLEECLSTGCRDSEHEGHKVCTSCCEGNICNLPLPRNETDATFA
(47)
(141)
dαN(i,i+3)
C
Lypd6b
MFKCFTCENAGDNYNCNRWAEDKWCPQNTQYCLTVHHFTSHGRSTSITKKCASRSECHFVGCHHSRDSEHTECRSCCEGMICNVELPTNHTNAVFA
(60)
(154)
dNN(i,i+2)
dαN(i,i+2)
dαβ(i,i+3)

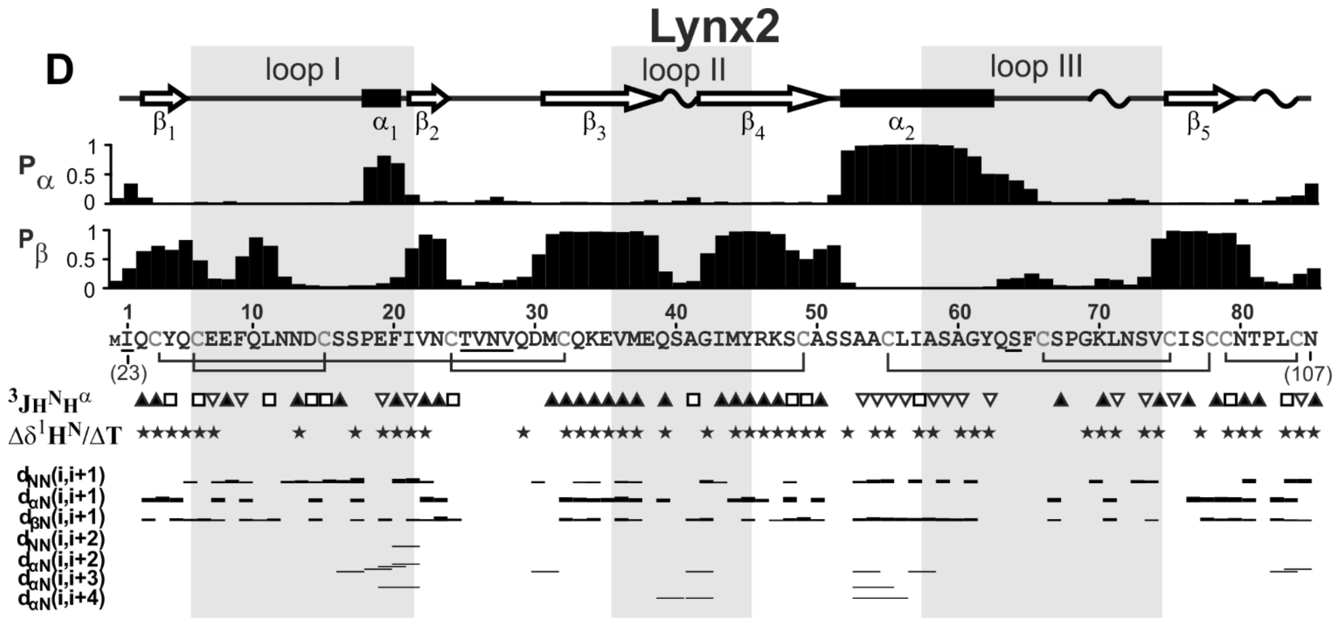

Figure 2. NMR data define the secondary structure and conformational heterogeneity of SLURP-1 (A), Lypd6 (B), Lypd6b (C), and Lynx2 (D). Data for *cis-* isomers of SLURP-1 and Lypd6 are shown by red symbols. From the top to the bottom of the panels.

Δδ, – net difference in $^{15}N^{H}$, $^{1}H^{N}$, and $^{1}H^{\alpha}$ chemical shifts ($\sqrt{(\Delta\delta_{HN})^2 + \left(\Delta\delta_{NH}/5\right)^2 + (\Delta\delta_{HA})^2}$) for the two isomeric forms of SLURP-1 and Lypd6.

Determined secondary structure. Tight β- and γ-turns are shown by wavy lines.

Pα and Pβ, – Probabilities of α-helix and β-structure formation calculated from ^{1}H, ^{13}C, and ^{15}N chemical shifts in the TALOS-N software.

Protein sequence. The loop regions and *C*-terminal fragments of Lypd6 and Lypd6b used for calculation of mean RMSD and S^2 values are highlighted by gray background. The numbers in brackets corresponds to the gene residue numbers.

$^{3}J_{HNH\alpha}$ coupling constants. The small (< 5.5 Hz), large (> 8.5 Hz), and medium (others) couplings are designated by open triangles, filled triangles and open squares, respectively.

$\Delta\delta^{1}H^{N}/\Delta T$, – temperature coefficients of amide protons. The black-filled stars denote amide protons with temperature gradients less than 4.5 ppb/K.

H/D$_{EX}$, – H-D exchange rates for HN protons measured at 37°C for SLURP-1 and 20°C for Lypd6. The black-filled, gray-filled, and open circles denote HN protons with slow (half-exchange time > 30 h), slow-intermediate (half-exchange time > 2 h) and intermediate (half-exchange time > 20 min) H/D$_{EX}$, respectively.

Local NOE contacts ($\boldsymbol{d_{xx}}$) are shown as usual.

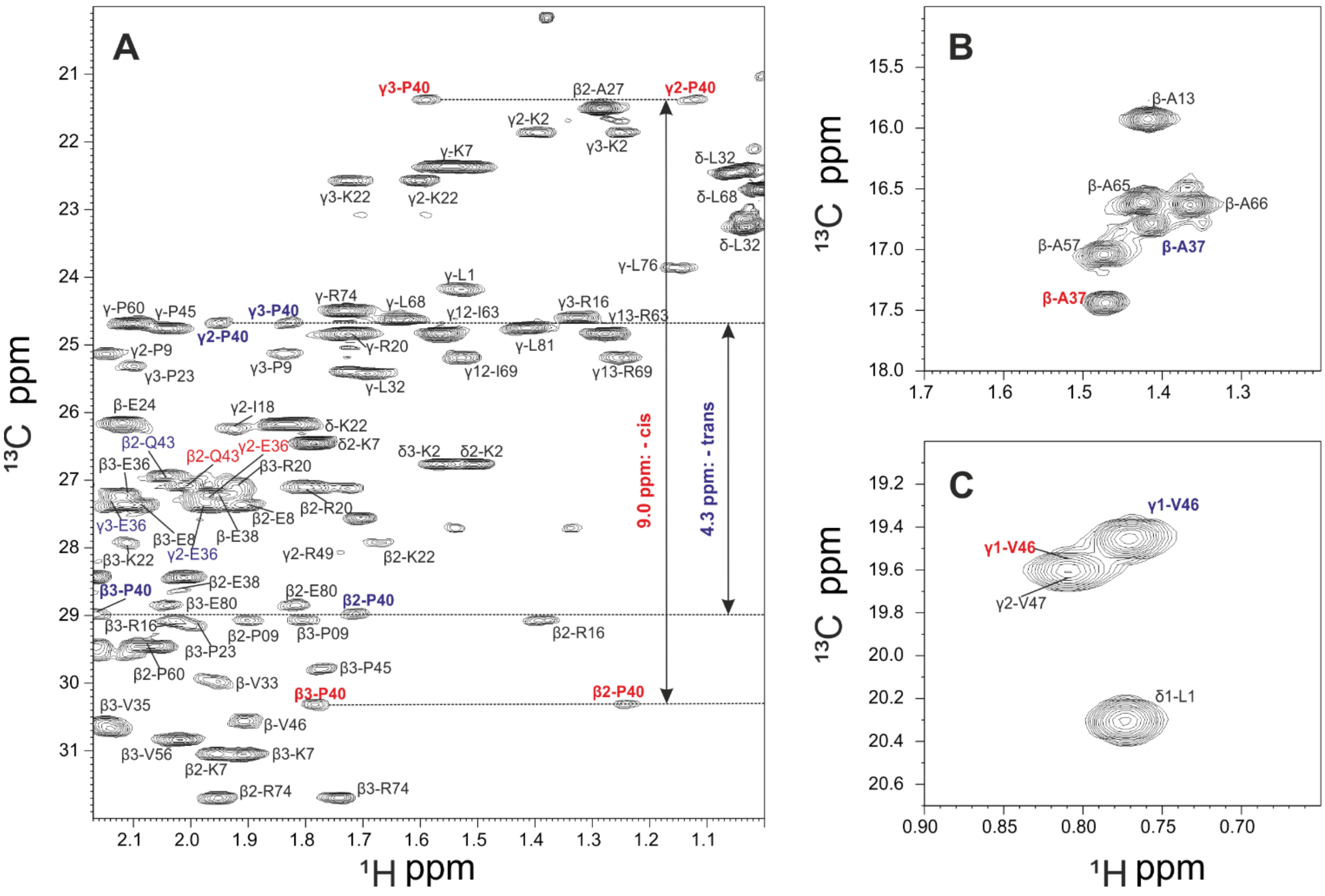

Figure 3. ^{13}C-HSQC spectrum of ^{13}C,^{15}N-labelled SLURP-1 illustrates conformational heterogeneity and Pro40 assignment. (A). Region of the proline signals. Difference in chemical shifts between $^{13}C^{\beta}$ and $^{13}C^{\gamma}$ nuclei in Pro40 confirms the assignment of the two forms to the *trans*- and *cis*- isomers of Tyr39–Pro40 peptide bond. (B, C). Fragments of methyl region of ^{13}C-HSQC spectrum. The signals of *trans*- and *cis*- Tyr39–Pro40 isomers are labeled by blue and red, respectively. Non-splitted signals belonging to both isomers are in black.

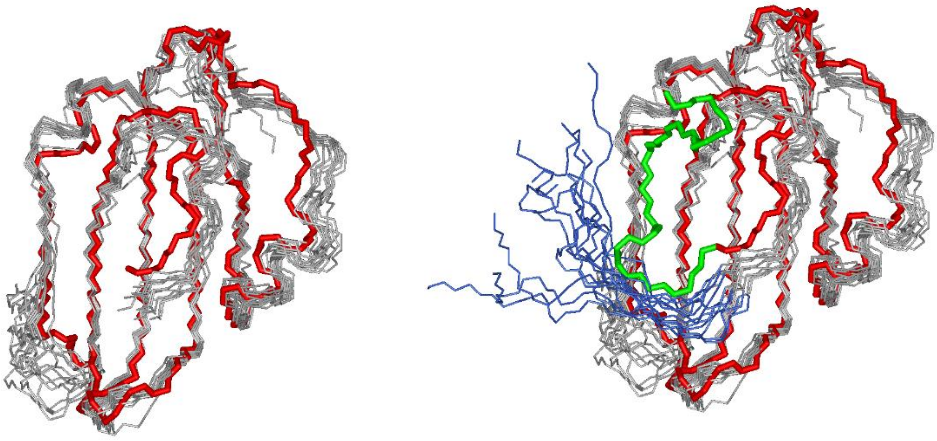

Figure 4. Comparison of the Lypd6 3D structures obtained by X-ray (Red, PDB 6GBI) and NMR (Grey, PDB 6IB6). Only backbone atoms are shown. The set of 20 best NMR structures is shown. Mobile *C*-terminal region (Pro85-Ala95) is omitted on the left panel and shown in blue (NMR structures) and green (X-ray structure) on the right panel.

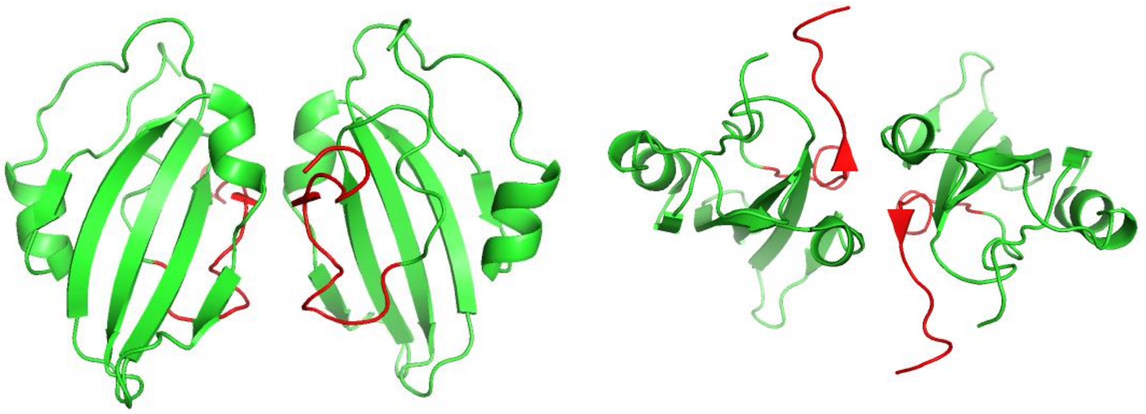

Figure 5. Contacts between neighboring asymmetric units in the crystal structure of Lypd6 (PDB 6GBI). *C*-terminal region Pro132-Leu147 (original numeration; Pro132 corresponds to Pro85 in this work) is shown in red.

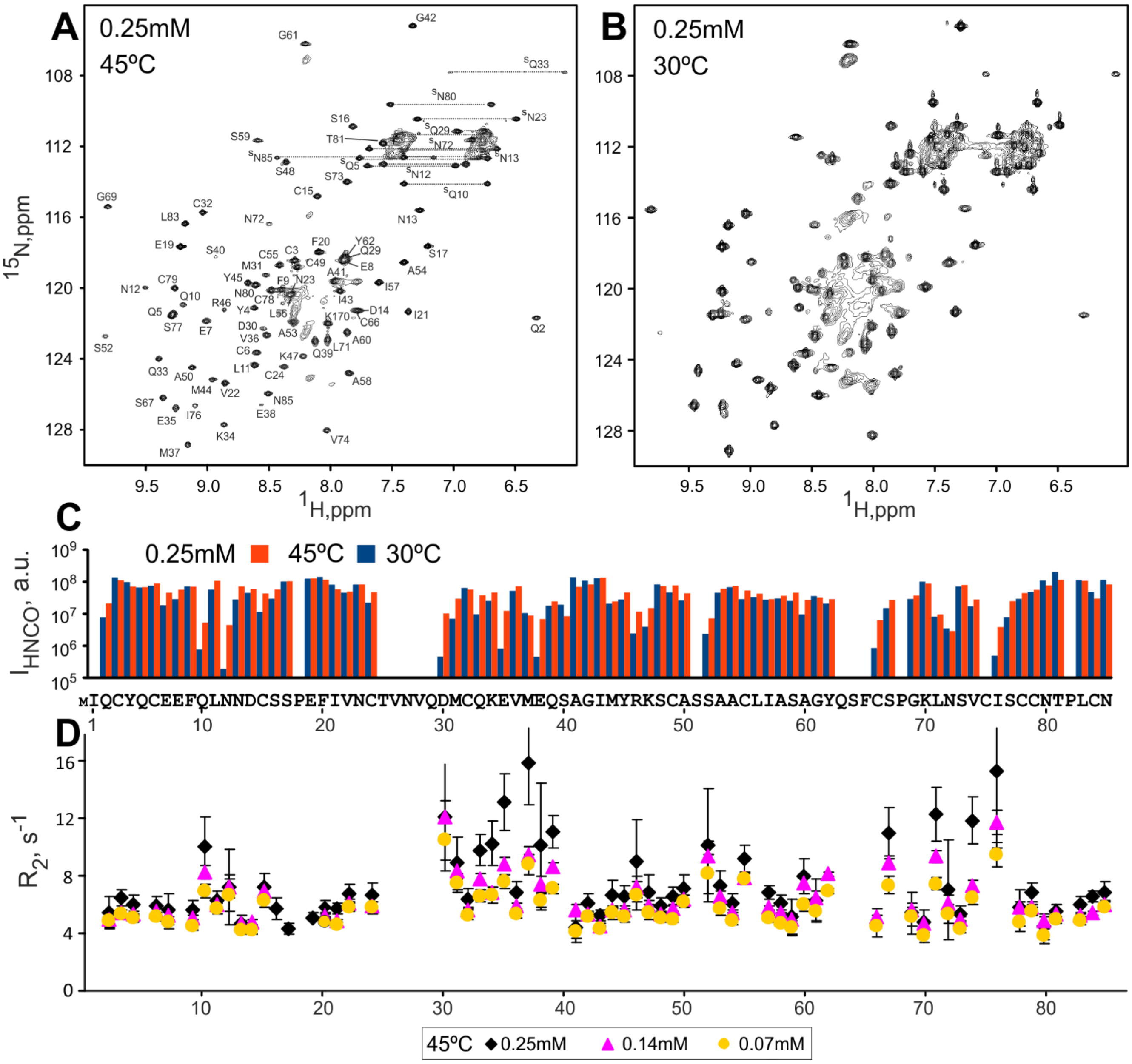

Figure 6. NMR spectral parameters of Lynx2 at different concentrations and temperature. (**A, B**). Comparison of the ^{15}N-HSQC spectra of 0.25 mM ^{13}C,^{15}N-labelled Lynx2 at 45°C (**A**) and 30°C (**B**). (**C**). Comparison of cross-peak intensities in the 3D HNCO spectrum of 0.25mM Lynx2 at 45°C and 30°C. (**D**). Transverse relaxation rates (R_2) of Lynx2 ^{15}N nuclei at different protein concentrations (60 MHz, 45°C, pH 6.8).

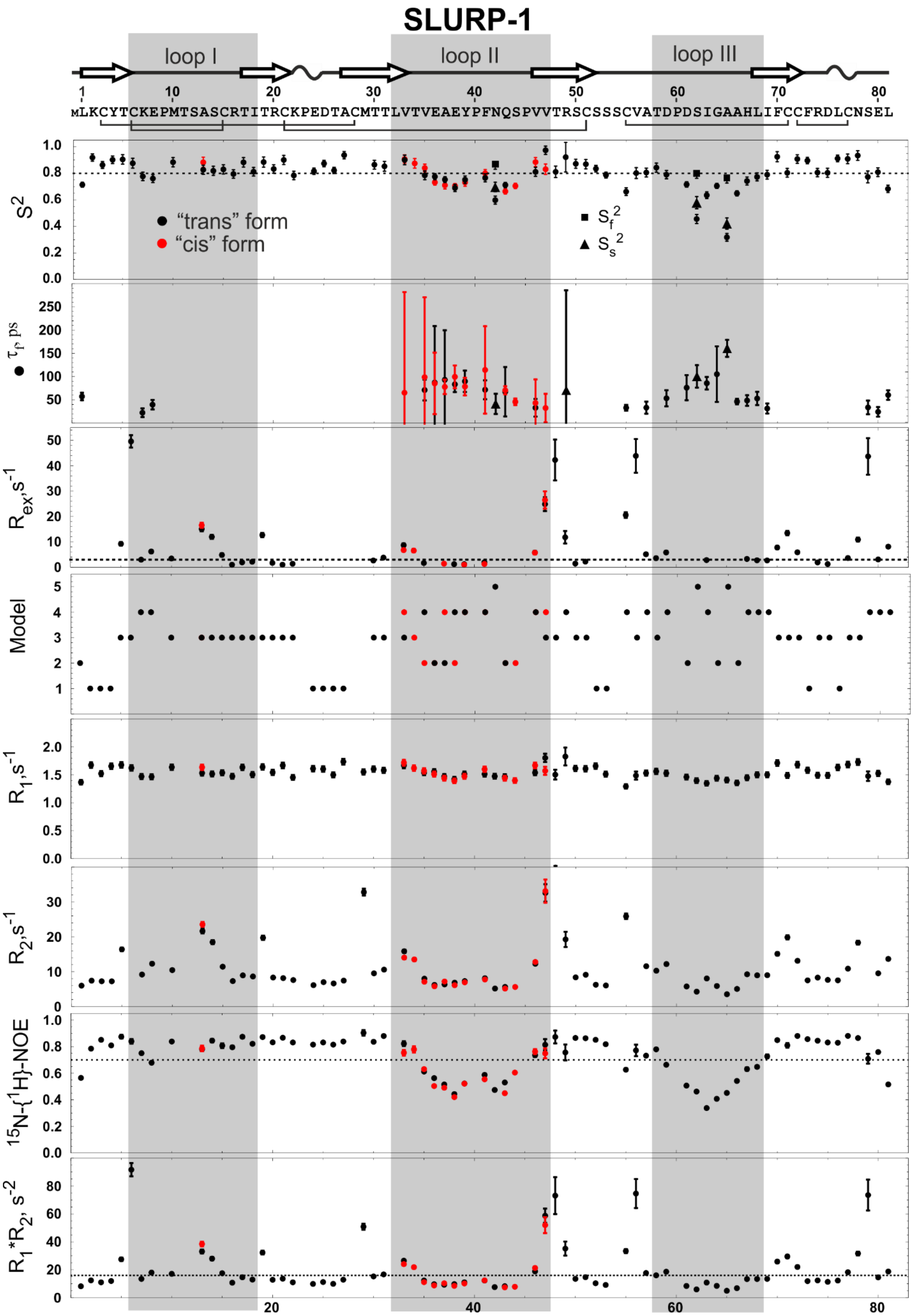
SLURP-1
loop I
loop II
loop III
MLKCYTCKEPMTSASCRTITRCKPEDTACMTTTLVTVEAEYPFNQSPVVTRSCSSSCVATDPDSIGAAHLIFCCFRDLCNSEL
S2
"trans" form
"cis" form
Sf2
Ss2
τf, ps
τs, ps
Rex, s-1
Model
R1, s-1
R2, s-1
15N-{1H}-NOE
R1*R2, s-2

Figure 7A.

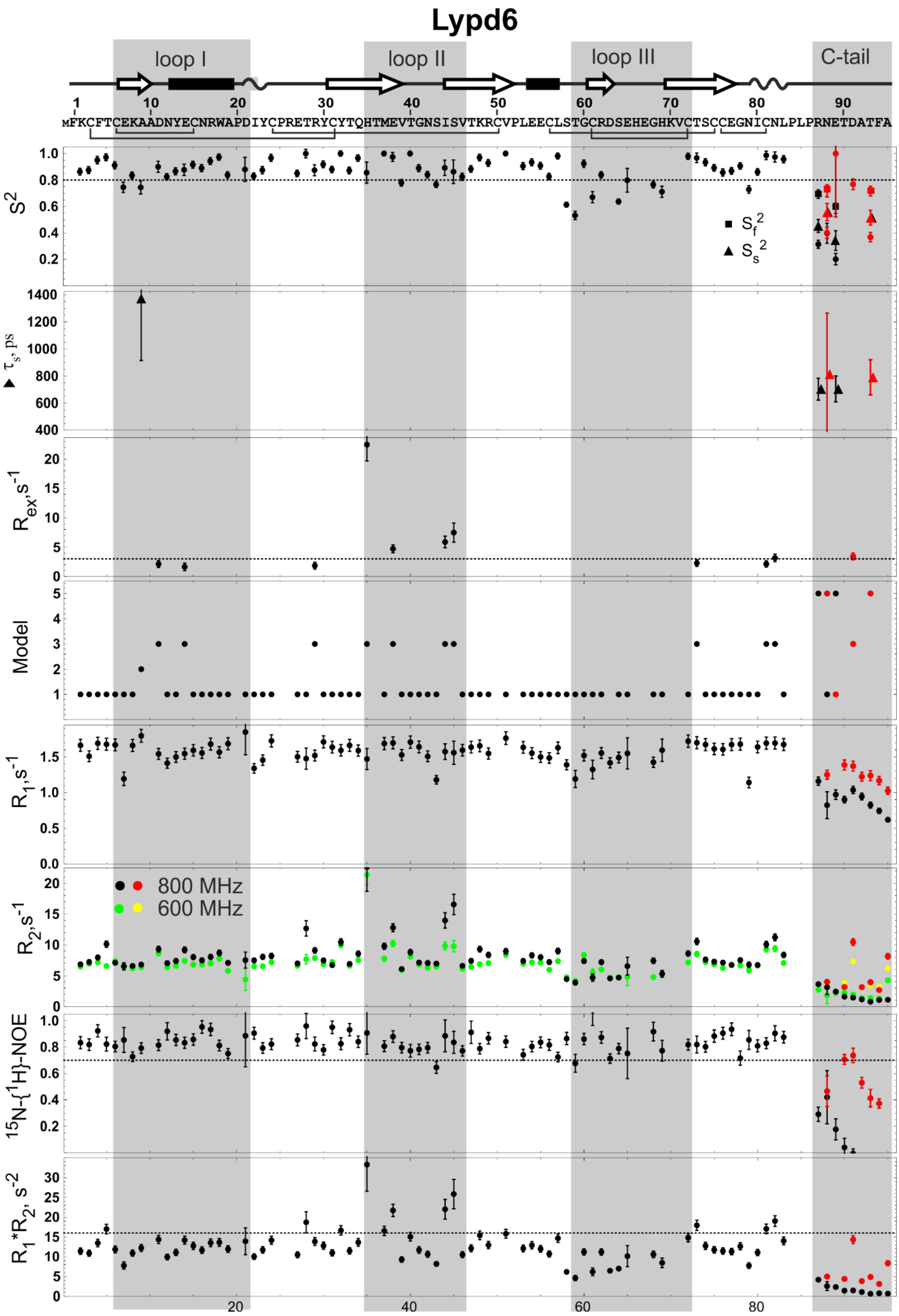
Lypd6
loop I
loop II
loop III
C-tail
MFKCFTCEKAADNYECNRWAPDIYCPRETRYCYTQHTMEVTGNSISVTKRCVPLEECLSTGCRDSEHEGHKVCTSCCEGNICNLPLPRNETDATFA
S2
τs, ps
Rex, s-1
Model
R1, s-1
R2, s-1
800 MHz
600 MHz
15N-{1H}-NOE
R1*R2, s-2

Figure 7B.

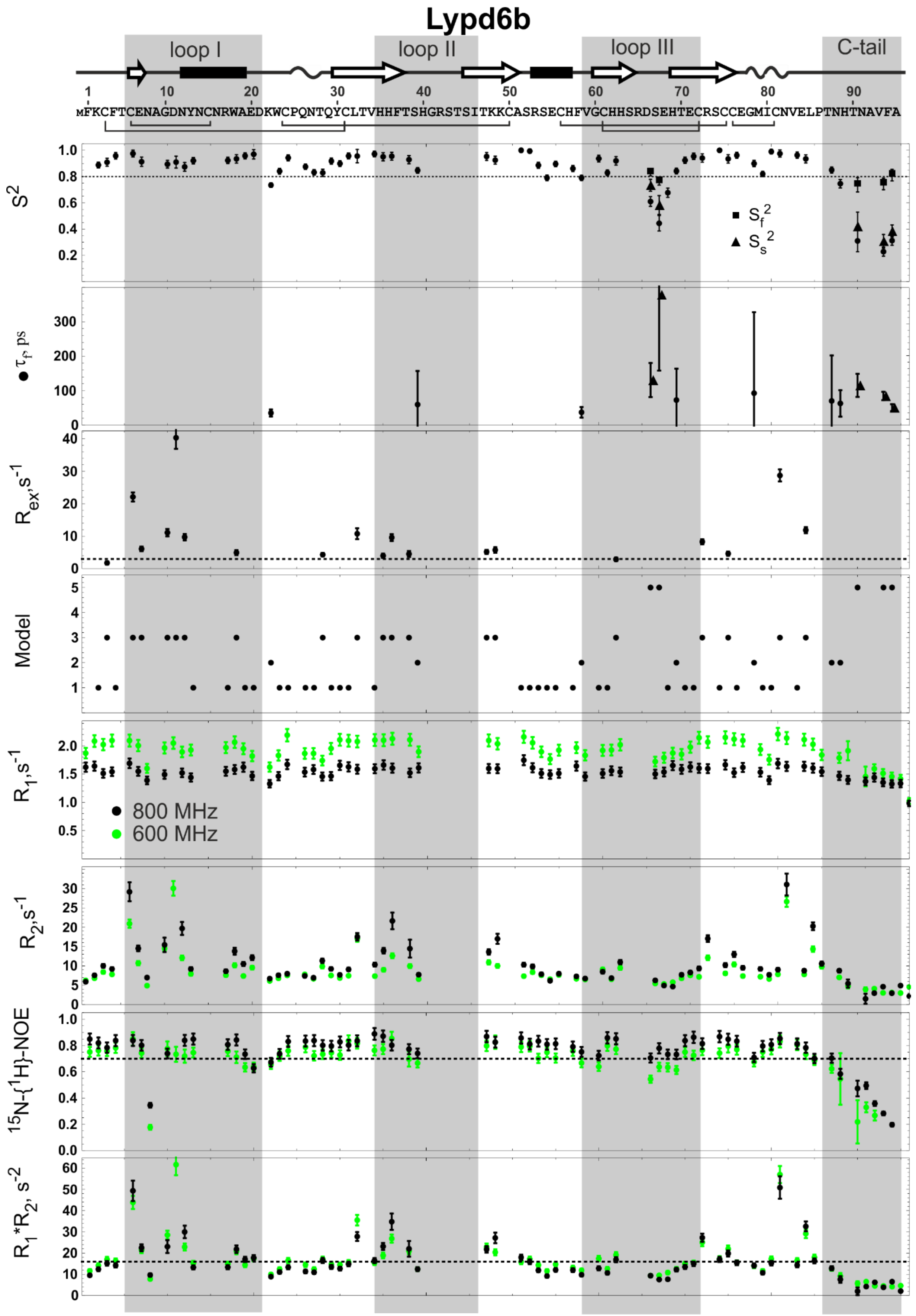
Lypd6b
loop I
loop II
loop III
C-tail
MFKCFTCENAGDNYNCNRWAEDKWCPQNTQYCLTVHHFTSHGRSTSITKKCASRSECHFVGCHHSRDSEHTECRSCCEGMICNVELPTNHTNAVFA
S2
τf, ps
τs, ps
Rex, s-1
Model
R1, s-1
800 MHz
600 MHz
R2, s-1
15N-{1H}-NOE
R1*R2, s-2

Figure 7.C.

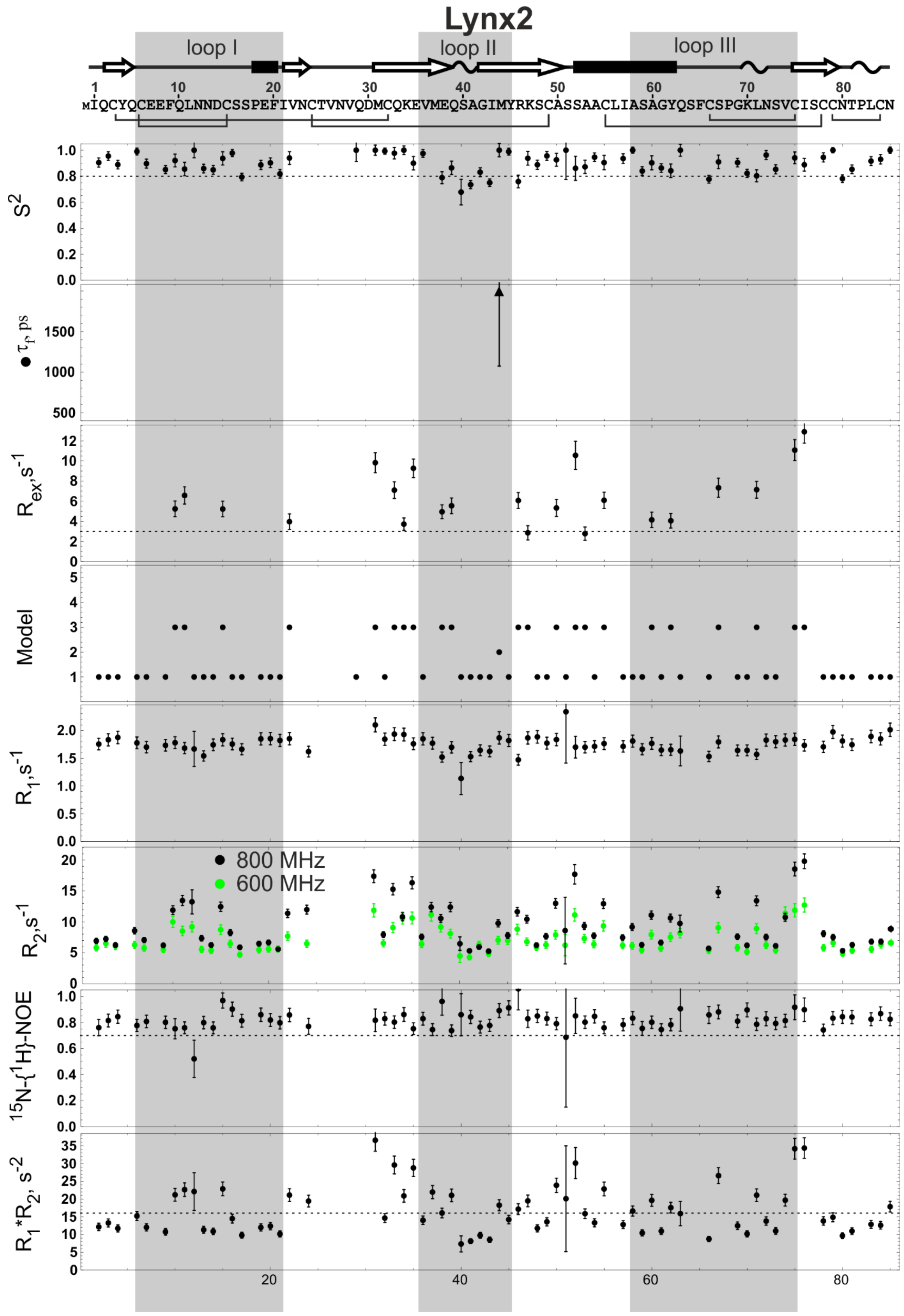
Lynx2
loop I
loop II
loop III
MIQCYQCEEFQLNNDCSSPEFIVNCTVNVQDMCQKEVMEQSAGIMYRKSCASSAACLIASAGYQSFCSPGKLNSVCISCCNTPLCN
S2
τf, ps
Rex, s-1
Model
R1, s-1
R2, s-1
800 MHz
600 MHz
15N-{1H}-NOE
R1*R2, s-2

Figure 7.D.

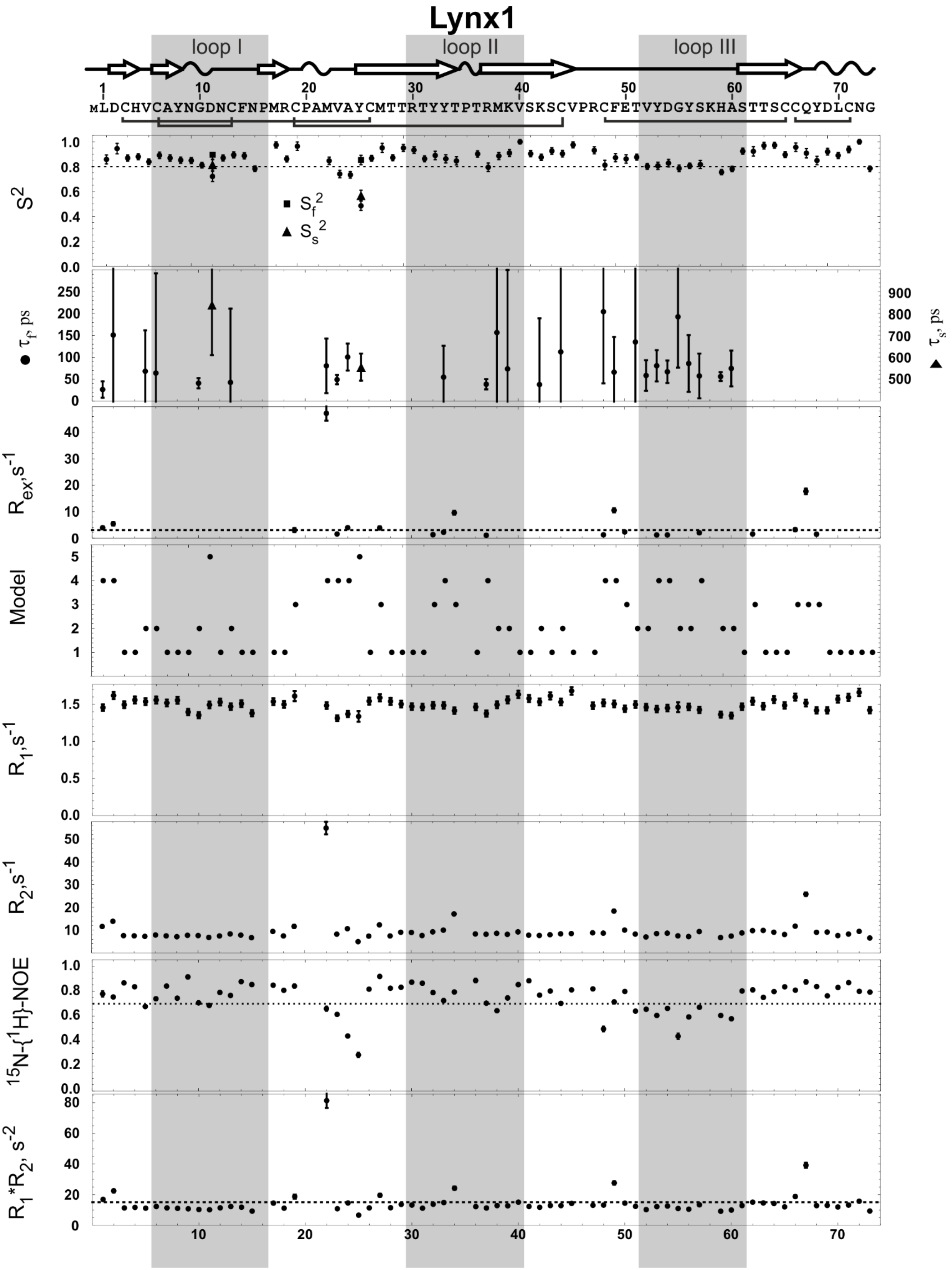

Lynx1
loop I
loop II
loop III
MLDCHVCAYNGDNCFNPMRCPAMVAYCMTTRTYYTPTRMKVSKSCVPRCFETVYDGYSKHASTTSCCQYDLCNG
S²
S_f²
S_s²
τ_f, ps
τ_s, ps
R_ex, s⁻¹
Model
R_1, s⁻¹
R_2, s⁻¹
¹⁵N-{¹H}-NOE
R_1*R_2, s⁻²

Figure 7E.

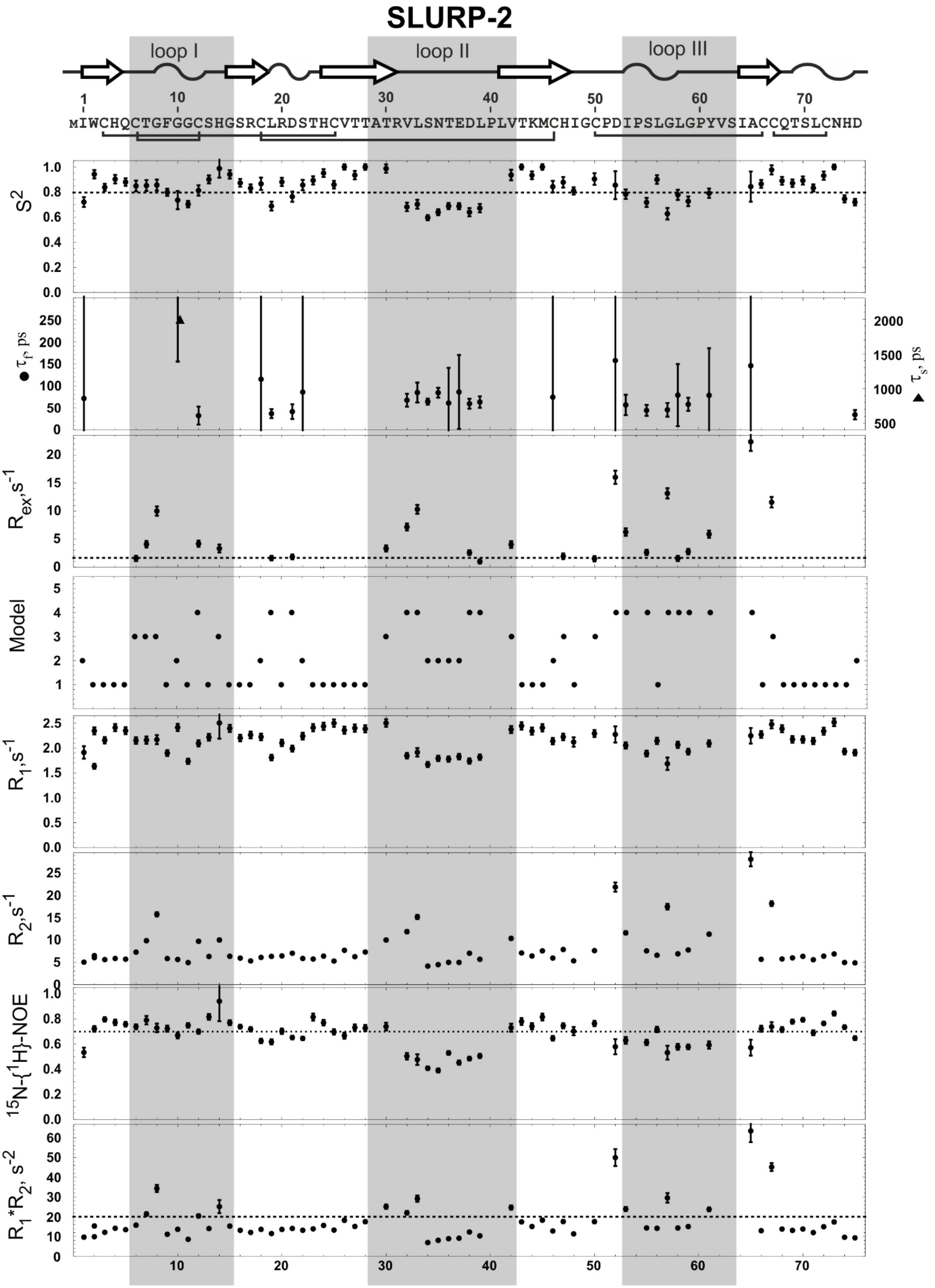
SLURP-2
loop I
loop II
loop III
MIWCHQCTGFGGCSHGSRCLRDSTHCVTTATRVLSNTEDLPLVTKMCHIGCPDIPSLGLGPYVSIACCQTSLCNHD
S2
τf, ps
τs, ps
Rex, s-1
Model
R1, s-1
R2, s-1
15N-{1H}-NOE
R1*R2, s-2

Figure 7F.

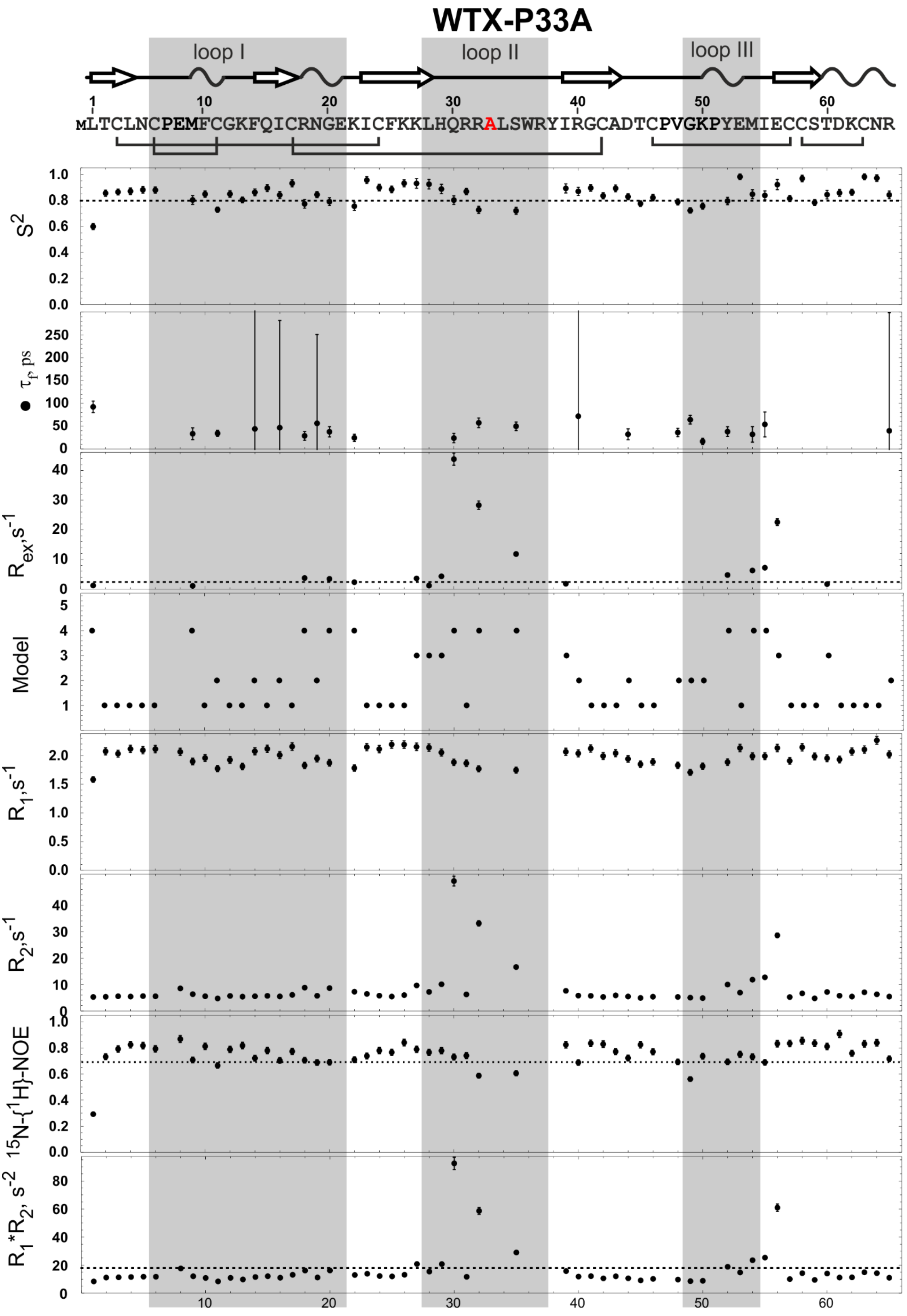
WTX-P33A
loop I
loop II
loop III
mLTCLNCPEMFCGKFQICRNGEKICFKKLHQRRALSWRYIRGCADTCPVGKPYEMIECCSTDKCNR
S2
τf, ps
Rex,s-1
Model
R1,s-1
R2,s-1
15N-{1H}-NOE
R1*R2, s-2

Figure 7G.

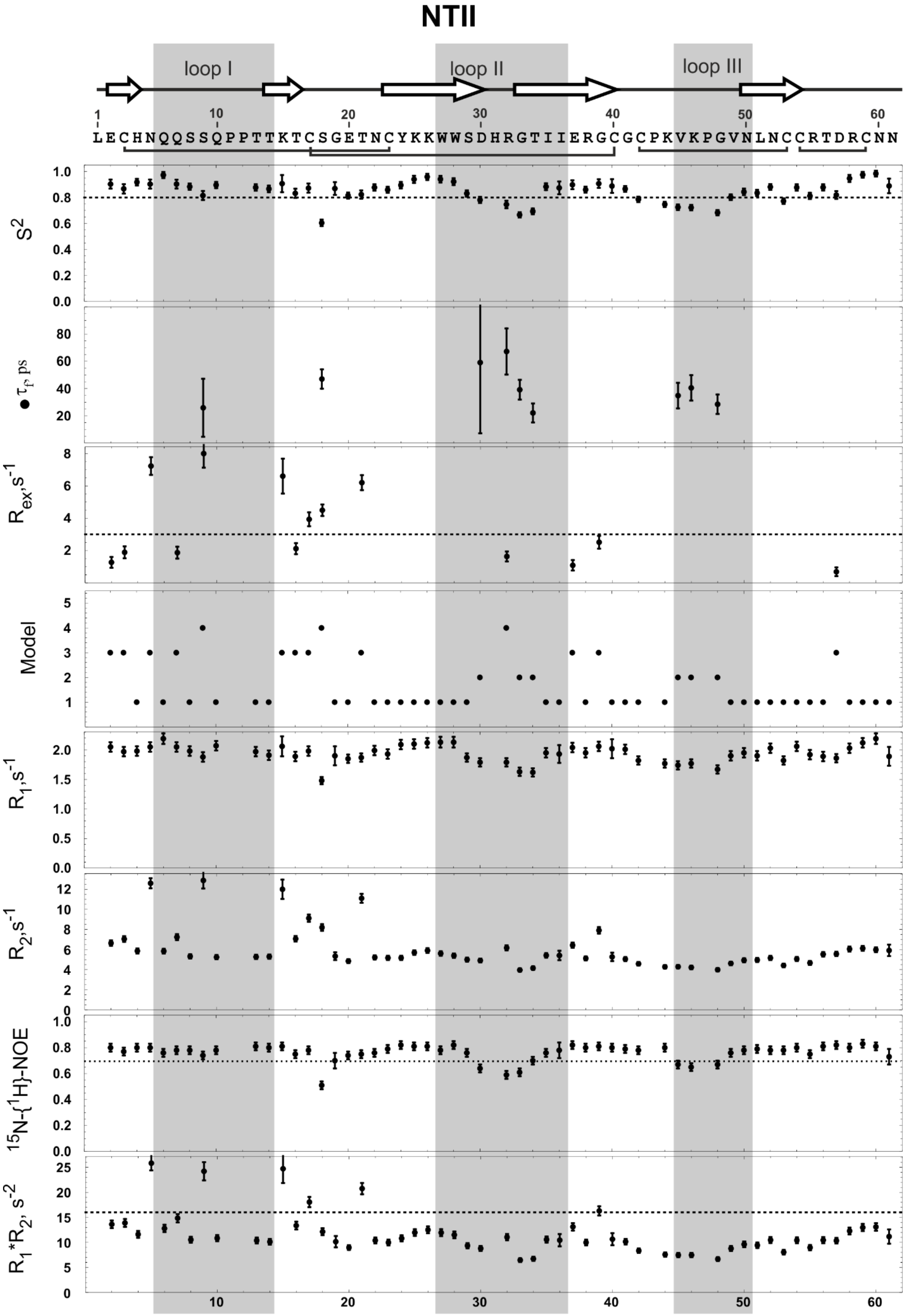

Figure 7H.

Figure 7. [15]N relaxation data and results of the 'model-free' analysis for SLURP-1 (A), Lypd6 (B), Lypd6b (C), Lynx2 (D), Lynx1 (E), SLURP-2 (F), WTX-P33A (G), and NTII (H). The experimental

conditions temperature, pH, NMR frequency are given in Table 1. The additional sets of data measured for Lypd6, Lypd6b, and Lynx2 at 600 MHz are shown by green and yellow symbols. The isotropic overall rotational diffusion model was used. The resulting overall rotational correlation times are collected in Table 2 (τ_R experimental). Model – the number of relaxation model assigned by the FastModelFree software. S^2 – squared values of the generalized order parameter. For model #5 $S^2 = S_f^2 \times S_s^2$, where S_f^2 and S_s^2 are squared order parameters for the fast and slow motions, respectively. S_f^2 and S_s^2 are shown by squares and triangles, were appropriate. τ_f and τ_s – effective correlation times for ps and ns backbone motions, respectively. R_{ex} – exchange contribution to the transverse relaxation rate. R_1 and R_2 – the values of longitudinal and transverse ^{15}N relaxation rates. ^{15}N-{^{1}H}-NOE - steady-state heteronuclear NOE. Residues displaying ^{15}N-{^{1}H}-NOE < 0.7 or $S^2 < 0.8$ are subjected to extensive motions in ps-ns timescale. To define the sites of high amplitude ps-ns mobility, we used thresholds $S^2 < 0.8$. Residues displaying $R_1 \cdot R_2 > TH1$ s^{-2} [Kneller JM, et al. *J Am Chem Soc*. (2002) **124**:1852-3] or $R_{EX} > TH2$ s^{-1} are subjected to exchange fluctuations in μs-s timescale. The *TH1* values are equal to 16, 18, and 20 s^{-2} for ^{15}N relaxation data measured at 800 (SLURP-1, Lypd6, Lypd6B, Lynx2, Lynx1, NTII), 700 (WTX-P33A), and 600 MHz (SLURP-2), respectively. To define the sites of high amplitude μs-ms mobility, we used *TH2* values equal to 3.0, 2.3, and 1.7 s^{-1} for ^{15}N relaxation data measured at 800, 700, and 600 MHz, respectively. Fragments used for calculation of RMSD and mean S^2 values are highlighted by grey background. Red and yellow symbols show the values for the *cis*-forms of SLURP-1 and Lypd6.

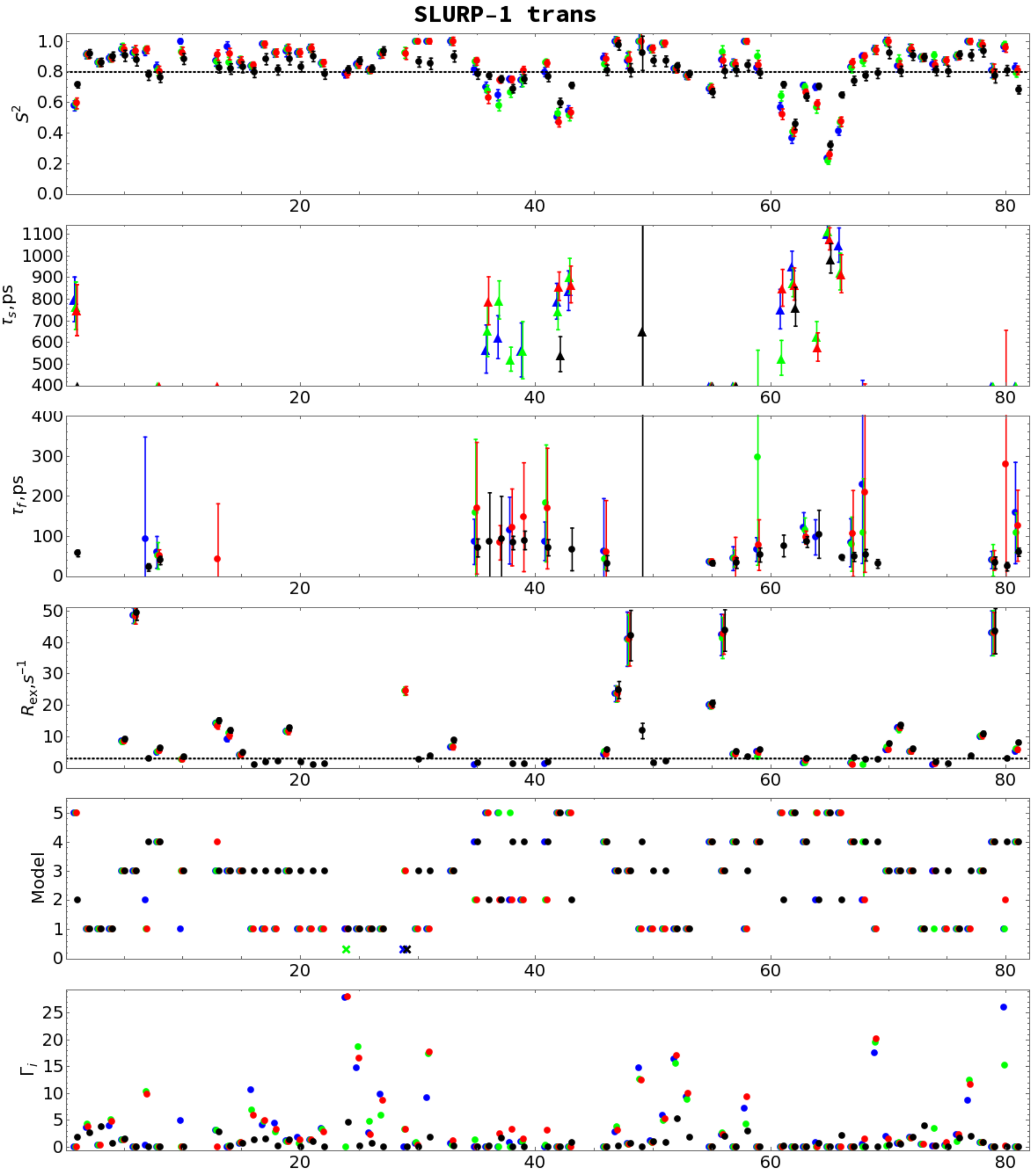

Figure 8A1.

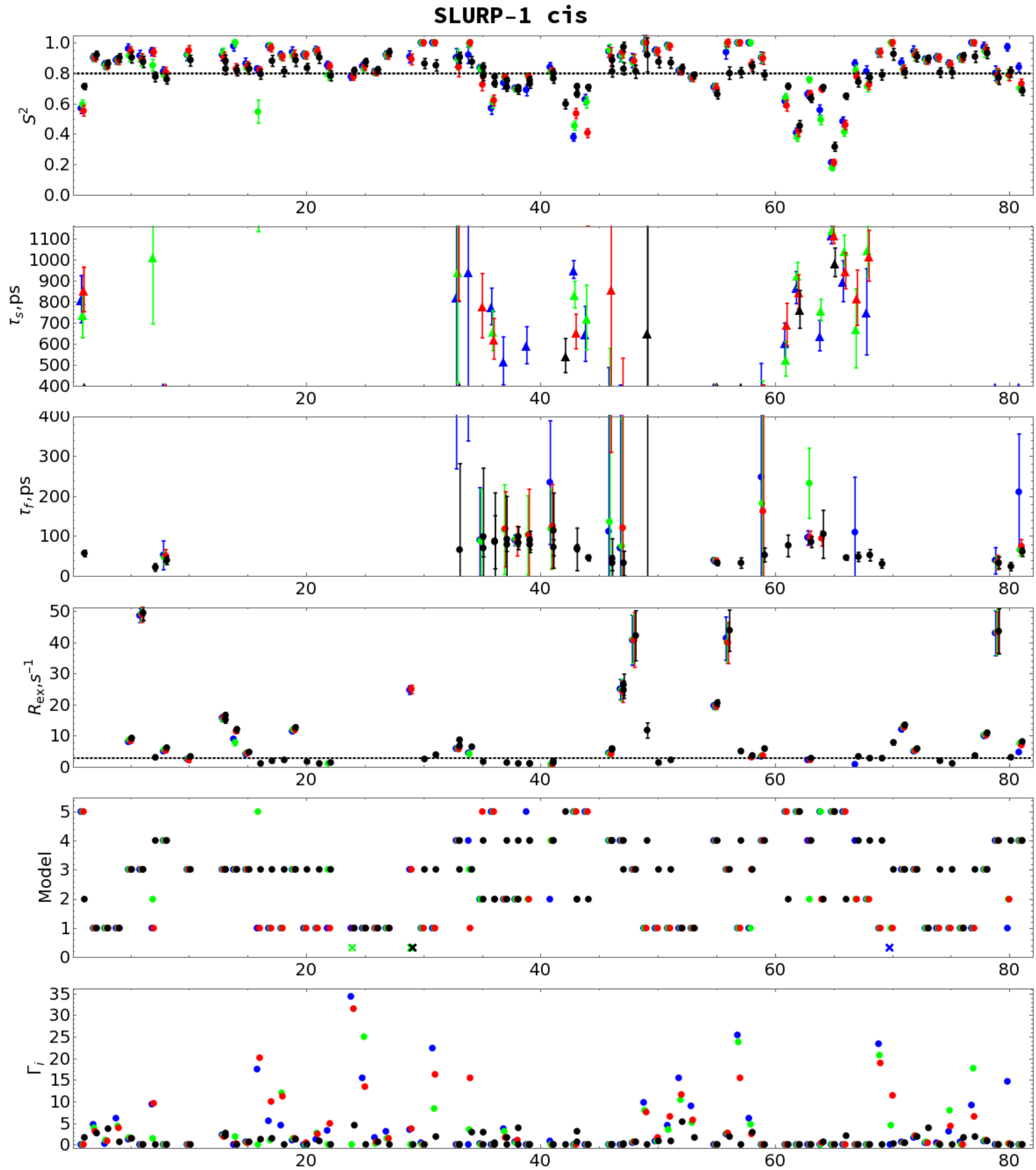

Figure 8A2.

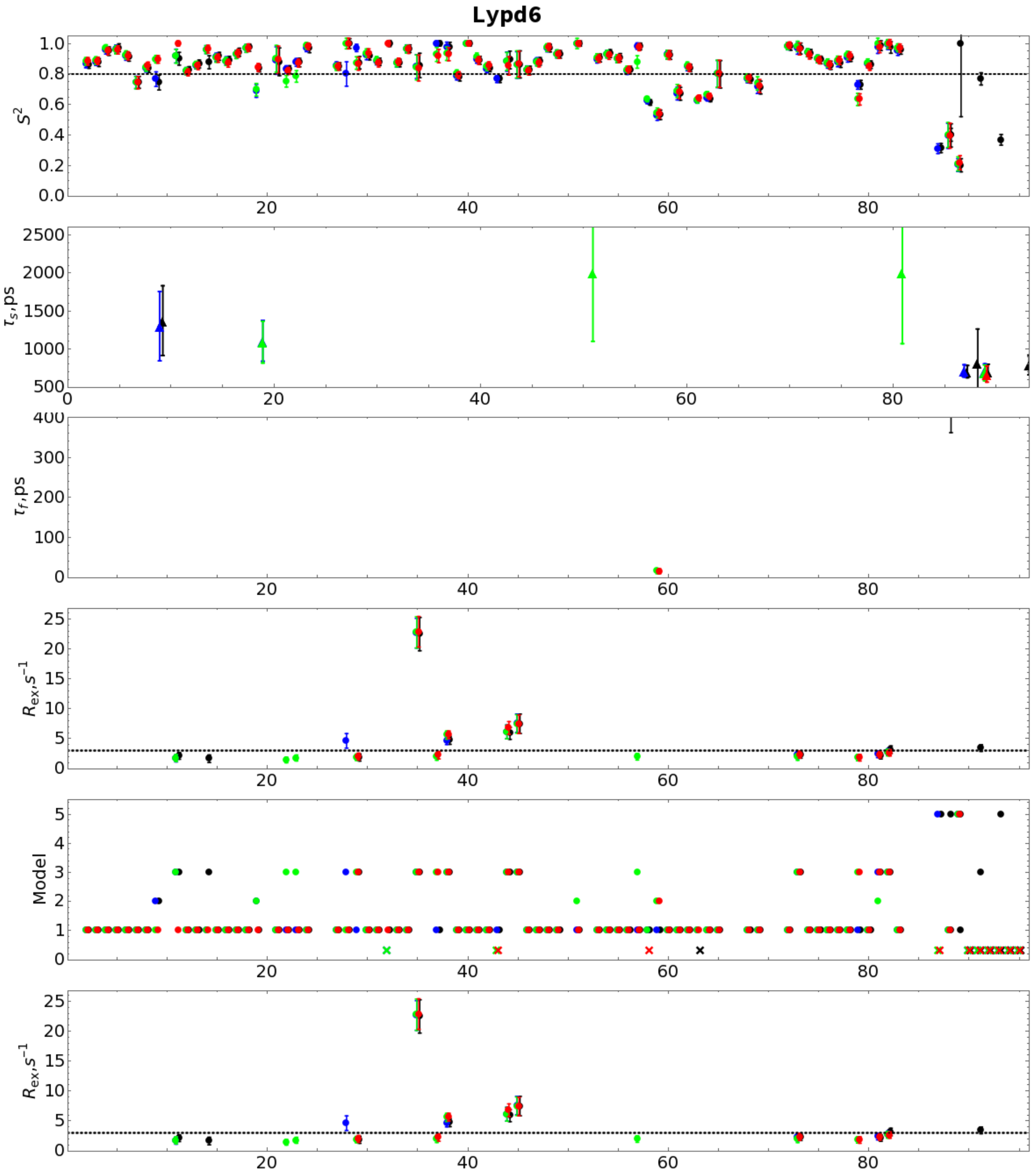
Lypd6
1.0
0.8
0.6
0.4
0.2
0.0
S^2
20
40
60
80
2500
2000
1500
1000
500
τ_s, ps
0
400
300
200
100
τ_f, ps
25
20
15
10
5
R_{ex}, s^{-1}
5
4
3
2
1
Model

Figure 8B.

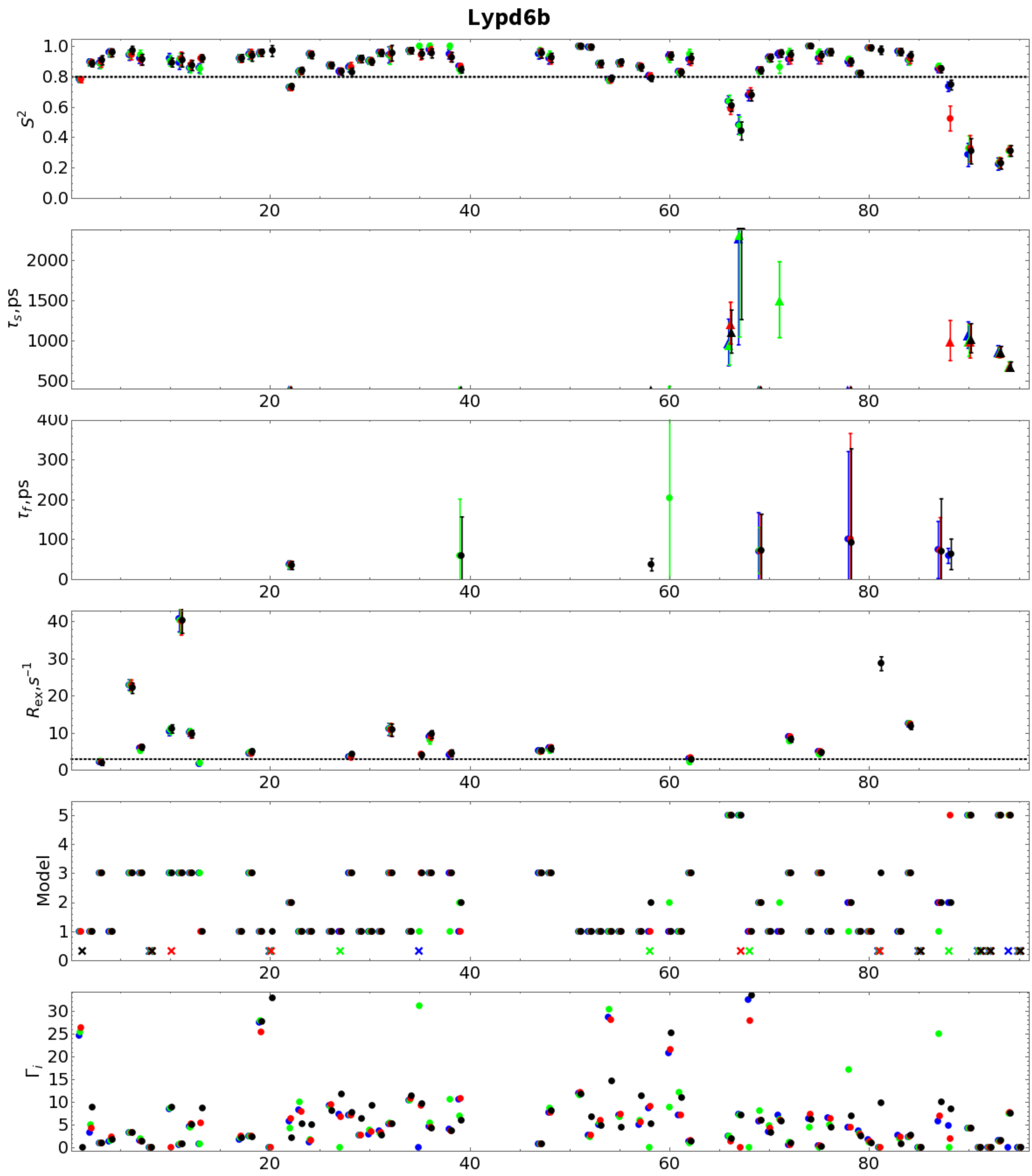

Figure 8C.

.

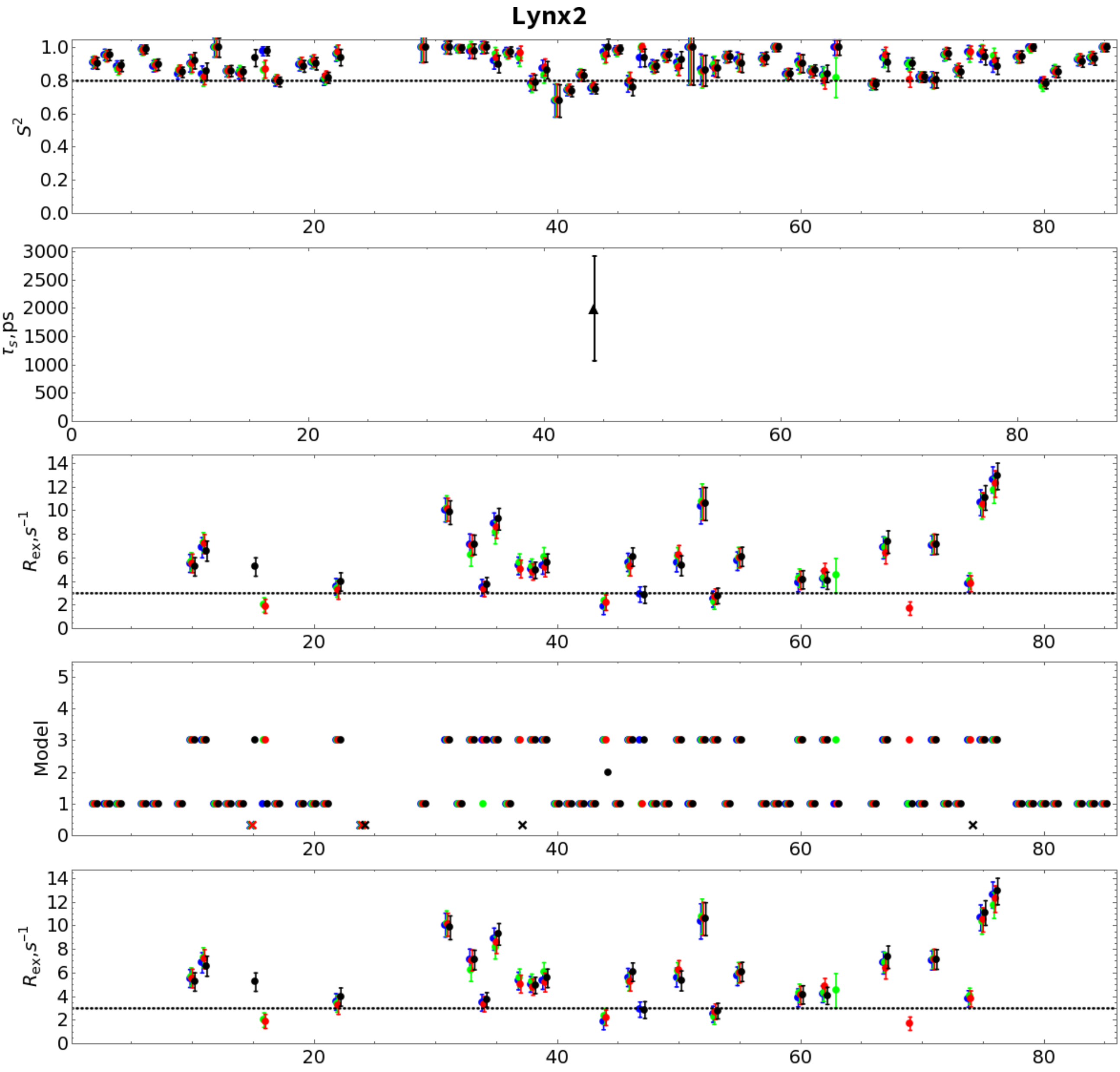

Figure 8D.

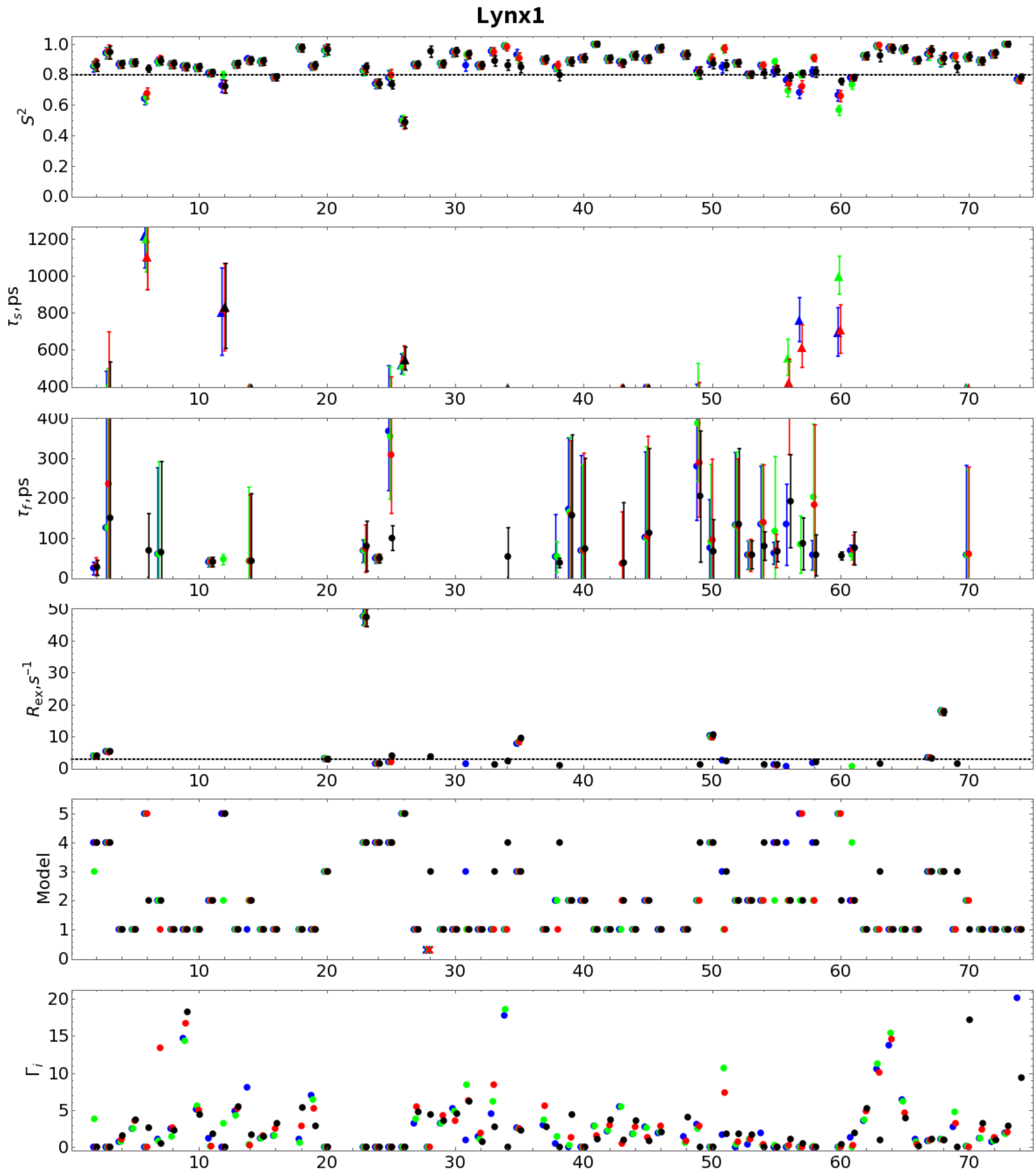

Figure 8E.

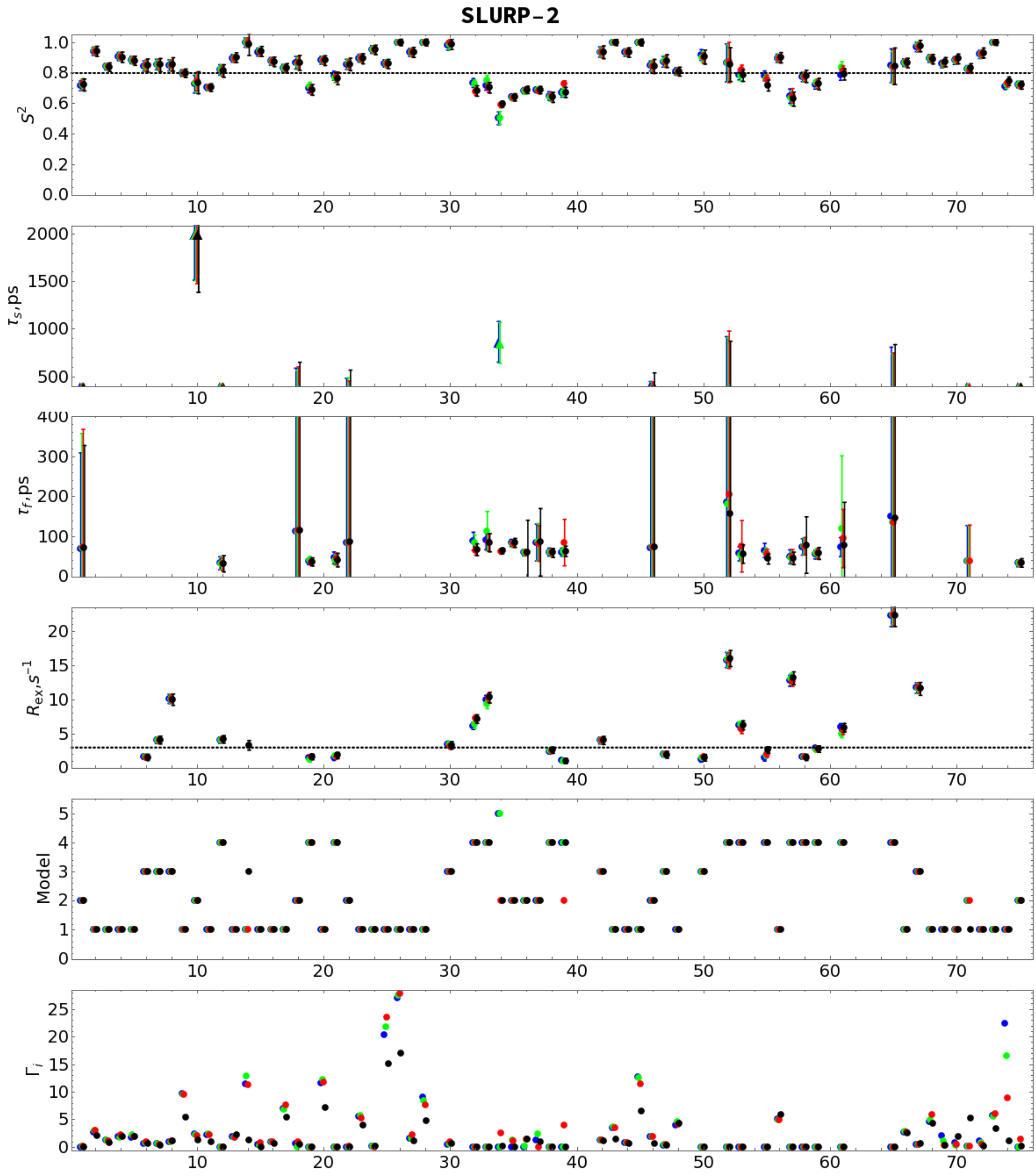

Figure 8F.

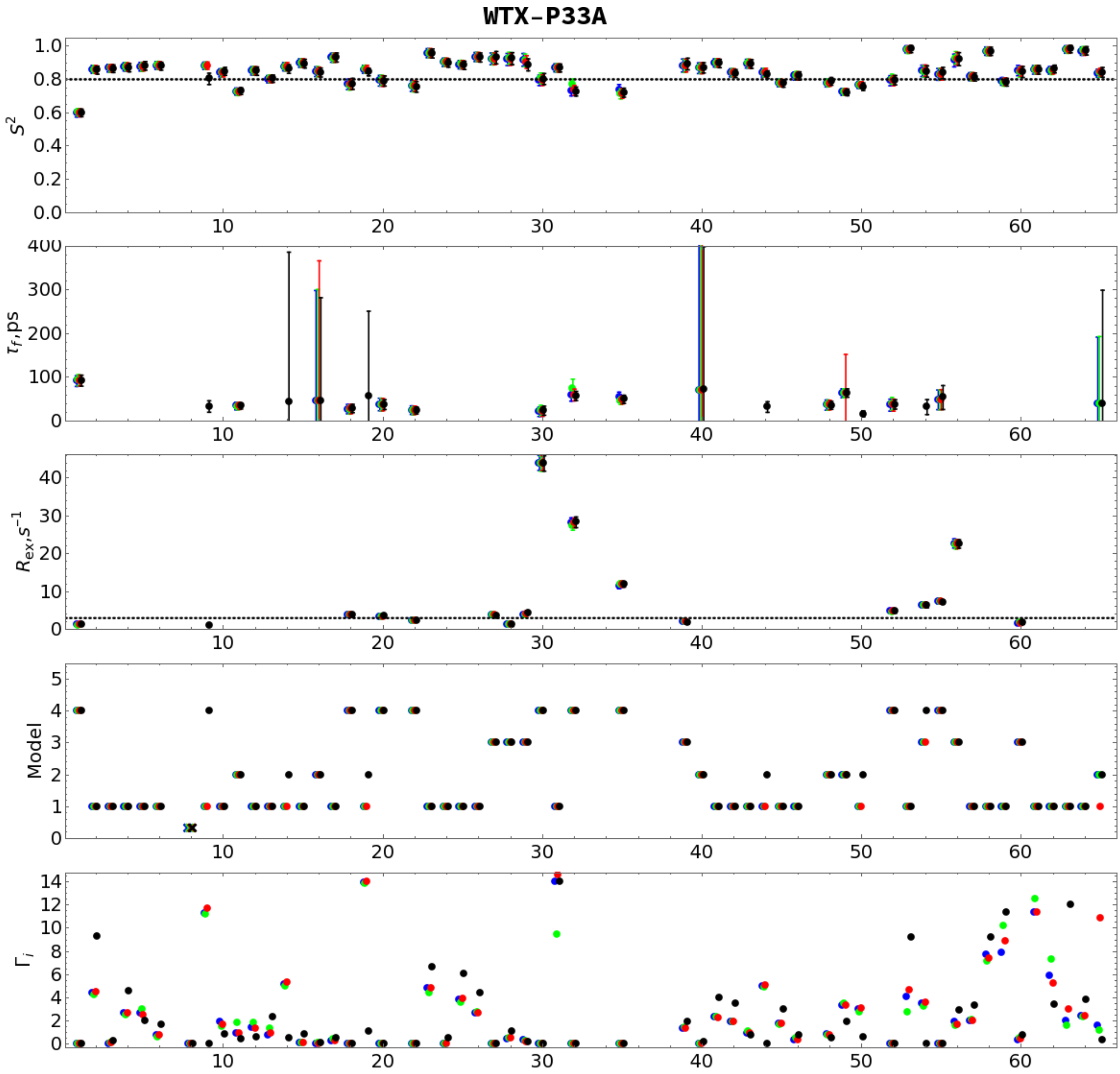

Figure 8G.

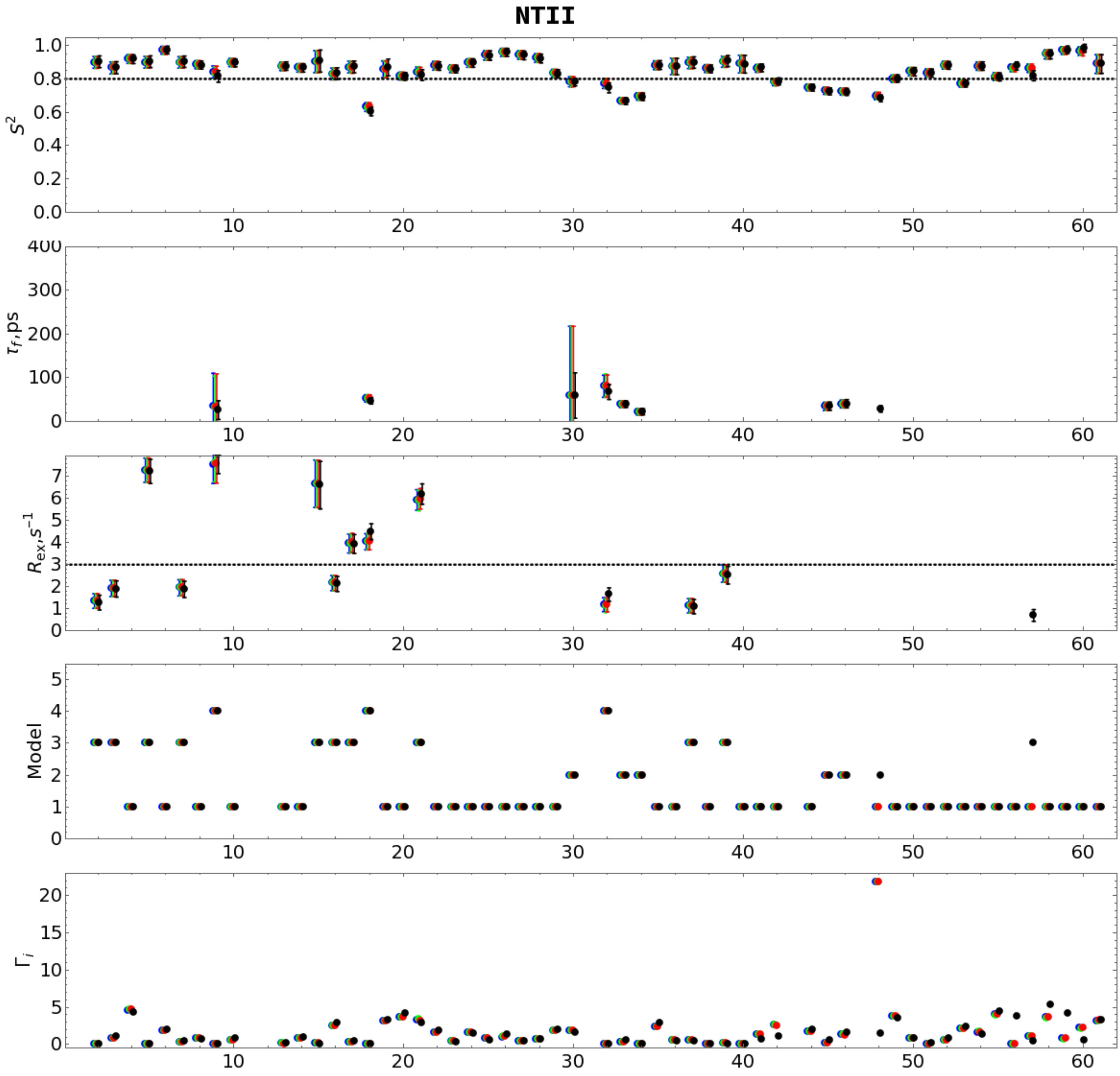

Figure 8H.

Figure 8. Choice of overall rotational diffusion model for the 'model-free' analysis of ^{15}N relaxation data for SLURP-1 (A1, A2), Lypd6 (B), Lypd6b (C), Lynx2 (D), Lynx1 (E), SLURP-2 (F), WTX-P33A (G), and NTII (H). For each protein the calculations were performed with isotropic rotational model (black dots) and with axially-symmetric model (oblate tensor) using three different conformers from the NMR structural ensemble (red, green and blue dots). Resulting overall rotational correlation times and tensor parameters are collected in Table 2 (experimental τ_R and anisotropy). Γ_i – sum-squared error of experimental data fitting by resulted model.

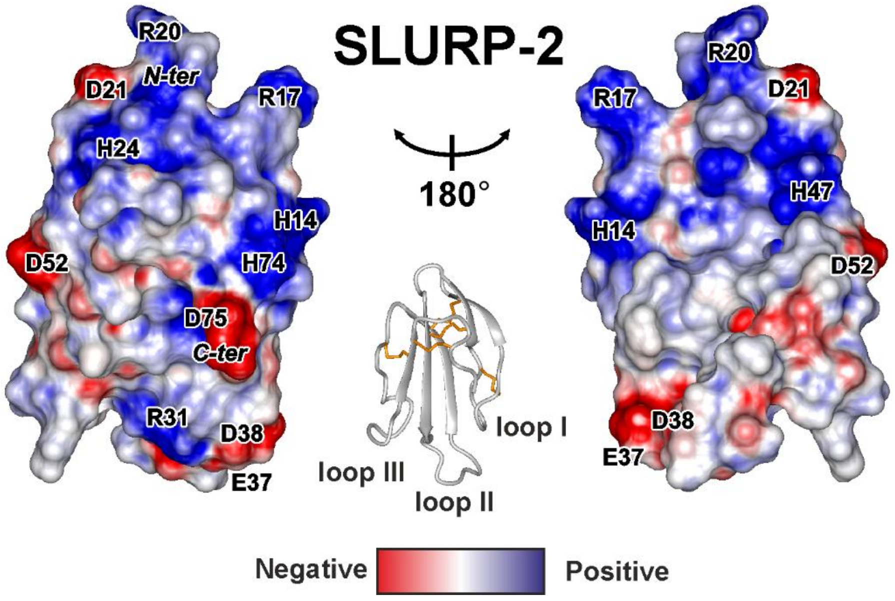

Figure 9. Two-sided view of electrostatic potential on the SLURP-2 molecular surface at weakly acidic pH. The sidechains were treated as positively charged. Red and blue areas denote negative and positive regions, respectively.
